# Improved anonymity preserving three-party mutual authentication key exchange protocol based on chaotic maps

**DOI:** 10.1371/journal.pone.0273664

**Published:** 2022-09-16

**Authors:** Kyong-Sok Pak, Mi-Hyang Kim, Song-Ho Pak, Chol-Man Ho

**Affiliations:** Faculty of Information Science, Kim Il Sung University, Pyongyang, Democratic People’s Republic of Korea; Civil Aviation University of China, CHINA

## Abstract

Three-party authentication key exchange is a protocol that allows two users to set up a session key for encrypted communication by the help of a trusted remote server. Providing user anonymity and mutual authentication in the authentication key exchange is important security requirements to protect users’ privacy and enhance its security performance. Recently Li proposed a chaotic maps-based authentication key exchange protocol which attempts to provide mutual authentication and user anonymity, but we found that there were some faults in the key exchange phase and password change phase of his scheme. We prove that Li’s scheme does not provide user anonymity and that the user’s privacy information is disclosed, and propose enhanced three-party authentication key exchange protocol that provides user anonymity and we analyse its security properties and verify its validity based on BAN logic and AVISPA tool.

## 1. Introduction

Authentication key exchange is one of the important issues to ensure the confidentiality of network security as a way of sharing the session key to perform encryption communication between communication parties in public network.

Researchers have done a lot of work on the two-party authentication key exchange (2PAKE) scheme (only two parties participate in key exchange) [1–5] and three-party authentication key exchange (3PAKE) scheme (except two communicating parties, the trusted third party server participates in key exchange) [6–43]. The main focus in authentication key exchange is to propose a clear authentication and a secure key exchange between participants. Key exchange is a process of setting up a session key to encrypt a message exchanged between participants and only two parties must share the key and the security of the key must be guaranteed. Typical encryption methods used for key exchange include secret-key encryption [26–43] and public-key encryption. Public-key encryption methods include in detail modular exponential operation -based schemes [6–13], elliptic curve encryption-based schemes [14, 15, 30–33, 34], chaotic maps-based schemes [16–25] and bilinear pairing-based schemes [34, 36]. User authentication is a key issue in authentication key exchange as a process of verifying whether a user is legal or not, where it is important to use authentication factor. Authentication factors include knowledge-based factors (e.g., registered passwords), ownership-based factors (e.g., smart card), biometric infrastructures (fingerprints, irises, etc.) [33]. According to the number of authentication factors, it is classified into single factor authentication [6–13, 16, 20, 34], two-factor authentication [14, 15, 26, 29, 38–42, 44], three-factor authentication [1, 3, 4, 24, 25, 28, 30, 32, 33, 37].

Recently, with the introduction of technologies such as peer-to-peer, cloud computing, wireless sensor network, and Internet of Things (IoT), researchers are further investigating 3PAKE.

### 1.1 Related work

Password-based authentication key exchange is a traditional method, and many researchers have proposed password-based authentication key exchange methods [6–13, 16, 20, 34]. However, several security disadvantages have been revealed in the authentication key exchange scheme using only passwords.

Tallapally [6] proposed a simple 3PAKE protocol based on password in wireless communication networks, however, Farash [7] has revealed that Lu’s scheme cannot detect online and offline password guessing attacks, and he has improved their scheme, but his scheme was also found to be vulnerable to offline password guessing attacks by Lu [8]. Lu proposed an improved scheme, but his scheme was still vulnerable to offline password guessing attacks [9].

Youn [10] proposed a 3PAKE protocol based on password and exponential operation. However, Heydari [11] pointed out that Youn’s scheme is vulnerable to user impersonate attack. Heydari proposed a modified 3PAKE protocol that overcame the limitations of the Youn’s scheme. However, his scheme also does not provide user anonymity because user’s identity is disclosed in the key exchange phase. Lin et al. [12] proposed verifier-based 3PAKE with low computational cost and transfer cost based on password and modulator exponential operation. However, Chiou [13] pointed out that Lin’s scheme does not provide anonymity and untraceability and is not computationally efficient, and proposed 3PAKE that provides anonymity and untraceability by implementing message encryption with long term key. However, since his scheme also performs key exchange [45] based on modular exponential operation, the computation is still not efficient.

Researchers used the Elliptic Curve Cryptography (ECC) [46] and Chebyshev Chaotic Maps (CCM) [47, 48] much more efficient compared to modular exponential operations. ECC encryption is fast because of its much smaller key length at the same encryption intensity compared to modular exponential operations. Chebyshev Chaotic Maps has a lower public parameter for encryption compared to ECC and is simple to implement and is convenient to apply in portable terminal-system environments.

Wu [14] proposed a key agreement scheme for mobile user roaming service in global mobility networks based on ECC. In his scheme user’s dynamic identity is updated in each session. However, Gupta [15] pointed out that Wu’s scheme fails to support untraceability and it has inefficient typo-detection.

Xie [16] proposed a 3PAKE protocol based on chaotic maps with user password. However, Lee [17] found that Xie’s scheme is vulnerable to offline password guessing attacks and does not provide user anonymity, and proposed a 3PAKE protocol that does not use passwords that overcome their shortcomings. In Lee’s scheme, user privacy is generated by combining the server’s secret key and the user’s identification, it is used to authenticate the corresponding user. However, Xie [18] found that Lee’s scheme is vulnerable to user impersonate attacks, and Jabbari [19] showed that Lee’s scheme is vulnerable to internal user impersonate attacks and does not provide anonymity.

Farash [20] proposed 3PAKE based on Chebyshev chaotic maps with user password. In his scheme user authentication verifier is generated by combining server privacy with user’s identifier and user’s password. However, Xie [21] and Li [22] found that Farashi’s scheme is capable of user impersonate attacks and offline password guessing attacks. Xie proposed an updated scheme based on chaotic maps overcoming the disadvantages of Farashi’s scheme. However, his scheme was also found by Lu [23] that offline password guessing attacks and user impersonate attacks are possible and user anonymity is not provided. Lu’s scheme encrypts a message with a secret key generated from the server’s public key based on the chaotic map to provide anonymity. However, his scheme has defects in protocol design [24].

To overcome the disadvantages of user authentication using passwords, researchers proposed 3PAKE protocols that combine smart card and biometric with user’s password to authenticate user.

In 2014, Xue [26] analysed Li [27] scheme that proposed a dynamic identifier-based 3PAKE in a multi-server environment and demonstrated that his scheme is vulnerable to attacks such as denial-of-service, internal attack, smart card attack, eavesdropping attack, masquerade attack. He also proposed an authentication key exchange scheme between a client and a service provider based on pseudo dynamic user identity using smart cards and user’s passwords in a multi-server environment. However, Gupta [28] found that his scheme is vulnerable to known password attacks, stolen smart card attacks and user impersonate attacks. In addition, Amin [29] also pointed out that Xue’s scheme does not provide anonymity, is vulnerable to offline password guessing attacks, privileged insider attacks, session key disclose attacks, and user impersonate attacks and has some defects in the authentication phase. Gupta proposed a hash function-based 3PAKE in a multi-service environment with user passwords and smart cards, but Tomar [30] demonstrated that his scheme is vulnerable to DoS attack, stolen smart card attack, user impersonate attacks, and does not provide perfect forward security.

Challa [32] also proposed a signature-based 3PAKE in IoT with password, smart card and biometric. However, Jia [33] pointed out that Challa’s scheme does not provide anonymity and untraceability and is vulnerable to user impersonate attacks, stolen smart card attacks, offline password guessing attacks, and attacks in the password change phase. Jia proposed a signature-based 3PAKE protocol that provides anonymity by updating Challa’s scheme. To provide anonymity, Jia used XOR operations and applied signature based on elliptic curve cryptography. Jia [34] proposed a 3PAKE scheme in fog-driven IoT based on Bilinear Pairing, whose scheme indicated by Ma [35] that it is computationally expensive because of Bilinear Pairing, and proposed a scheme that does not use Bilinear Pairing. Reddicherla [36] also proposed authentication key exchange scheme in Heterogeneous network based on Bilinear Pairing, but it is also not efficient because of the high computational cost.

Researchers also proposed key exchange scheme based on secret-key encryption without public-key encryption to implement 3PAKE in a portable terminal environment with narrow bandwidth and limited storage capacity, such as wireless communication environment or IoT. Key exchange based on secret-key encryption is much more advantageous in terms of computational cost because it does not use high-computational public-key encryption.

Chuang [37] proposed a 3PAKE scheme that provides anonymity with password, smart card and biometric in a multi-service environment. He proposed an authentication key exchange scheme that provides anonymity without public-key encryption in protocol design. However, his scheme was found by Amin [29] to be vulnerable to user impersonate attacks and vulnerable to session key disclose attacks. Amin analysed the disadvantages of the scheme proposed by Xue [26] and Chuang [37] and proposed an improved lightweight authentication scheme. His scheme provided anonymity using smart cards and passwords without public key encryption.

In 2018, Wei [38] also proposed a 3PAKE protocol that provides anonymity without public-key cryptography to reduce computational cost. In 2019, Yang [39] proposed a lightweight 3PAKE protocol that provides perfect forward security using only XOR and hash functions in a WSN environment.

### 1.2 Motivation and our contribution

The authentication scheme with password, smart card and biometric is effective in systems that require high security performance. However, most schemes using smart cards are vulnerable to stolen smart card attacks [26, 28, 32], and most schemes are vulnerable to some known attacks.

It is still a challenge for researchers to design protocols that are secure against various attacks in various environments while providing anonymity and untraceability. Many schemes attempted to provide anonymity and traceability, but failed [12, 14, 17, 20, 23, 32, 43].

Recently, in 2018, Li [38] proposed a chaotic maps-based 3-PAKE that provides anonymity with password and smart cards. In his scheme, users share user’s credentials related to user’s identity, user’s password and server’s secret with the server, and chaotic maps is used for exchanging session key. He also used modulo square operations and square root operations based on Chinese Remainder Theorem to encrypt the message providing anonymity and untraceability. However, there are drawbacks in his protocol.

We have analysed the disadvantages of the Li’s scheme and demonstrated that the user’s authentication verifier is disclosed by an internal attacker, providing anonymity is failed and that the password modification is not successful by blocking attacks in the password change phase. We design an enhanced 3PAKE protocol that overcomes several security disadvantages of Li’s scheme, and is resistant to various attacks. In this paper, we propose a strong mutual authentication between server and users to overcome insider attacks, and a re-registration phase that allows users to re-register without altering their identity. Then, we analyse the security properties of our scheme and verify its validity using Ban-Logic [49] and AVISPA [50] tools and show the results of comparative analysis with previous works.

## 2. Preliminaries

This section describes Chebyshev chaotic maps and Bio-hashing functions.

### 2.1 Chebyshev polynomials

Chebyshev polynomial *T*_*n*_(*α*) is defined as follows [47].


$$T_{n}(\alpha )=cos(n\cdot arcos(\alpha )),\alpha \in \left[-1,1\right],n\in N$$

Chebyshev polynomials satisfy the following recursive relationship [47].


$$T_{n}(\alpha )=2\alpha \cdot T_{n-}{}_{1}(\alpha )–T_{n-}{}_{2}(\alpha )(n>2),$$

$$T_{0}(\alpha )=1,T_{1}(\alpha )=\alpha$$

### 2.2 The property of Chebyshev polynomials

Chebyshev polynomials have the following two properties [47, 48].

Chaotic property: When *n*>1, Chebyshev polynomial map *T*_*n*_(*α*):[-1,1]→[-1,1] of degree n is a chaotic map with its invariant density $f^{*}(\alpha )=\frac{1}{\pi \sqrt{1-\alpha^{2}}}$, for positive Lyapunov exponent *ln*(*n*) > 0.

Semi-group property: For *u*, *v* ∈*N* and any *α*∈ [-1,1], *T*_*u*_ (*T*_*v*_(*α*)) = *T*_*uv*_(*α*) = *T*_*v*_(*T*_*u*_(*α*)).

### 2.3 Enhanced Chebyshev polynomials

The semi-group property holds for Chebyshev polynomials on the interval (-∞, +∞), which can enhance the property as follows [48]:

$$T_{n}(\alpha )=2\alpha \cdot T_{n-}{}_{1}(\alpha )–T_{n-}{}_{2}(\alpha )\;mod\;p(n\geq 2,\alpha \in \left(-\infty ,+\infty \right),p\;\text{is}\;\text{a}\;\text{large}\;\text{prime}\;\text{number}),$$

$$T_{u}(T_{v}(\alpha ))=T_{uv}(\alpha )=T_{v}(T_{u}(\alpha ))mod\;p(u,v\in N).$$

### 2.4 Computational problems based on Chebyshev polynomials

CDLP (Chaotic maps-based Discrete Logarithm problem): For given two real numbers *α* and *β*, it is infeasible to find the integer *n* by any polynomial time bounded algorithm, where *β* = *T*_*n*_(*α*) *mod p* [48].

CDHP (Chaotic maps-based Diffie-Hellman problem): For given three elements *α*, *T*_*m*_(*α*) *mod p* and *T*_*n*_(*α*) *mod p*, it is infeasible to compute the value *T*_*mn*_(*α*) *mod p* by any polynomial time bounded algorithm [48].

### 2.5 Bio-hashing and Fuzzy Extractor function

Biometric indicators have an advantage over traditional user identification methods, because these have some inherent attributes that cannot be easily shared and every person has unique biometric-attributes [51]. Generally, imprint biometric characteristics (face, fingerprint, palm-print etc.) may not be exactly same at each time since it might be change at some environment. To solve this problem, Lumini et al. [52] proposed and updated Bio-hashing, which is used to map a user’s biometric features to a user-specific random vectors. Recently many researchers [3, 24] have proposed authentication key exchange schemes based on Bio-Hashing.

Dodis et al. [53] proposed a scheme based on Fuzzy Extractor, which consists of two functions (*Rep*, *Gen*). The function *Gen* extracts biometric input *B* and outputs a nearly random binary string *R* and an auxiliary binary string *P*. Then function *Rep* recovers *R* with the corresponding auxiliary string *P* and biometric B*. If *dist* (*B*, B*) ≦ *t* and *Gen*(*B*) -> <*R*, *P*>, then we have *Rep* (B*, *P*) = *R*. Fuzzy Extractor is also used in many authentication schemes [1, 3, 4, 30, 32, 33].

### 2.6 Adversary model

For the security analysis of authentication protocols, several adversary models have been proposed, such as Dolev-Yao adversary model [54], side-channel technology [55], password guessing attack [56], and insider attacker model [4, 33].

The Dolev-Yao attacker model defines the ability of an attacker in the public channel, and the side-channel technology enables an attacker to extract data stored in a smart card based on reverse engineering and power analysis [57, 58]. Also, the password guessing attack enables an attacker to guess a password from the information related to the user’s password under the premise that the entropy of the password is low. An insider attacker is a legitimate user in the system and performs malicious actions.

In this subsection, the adversary model for security analysis of the previous work and the proposed scheme is described as follows.

1. An adversary can eavesdrop, modify, remove, block, and retransmit all messages sent on the public channel [54] and cannot access messages sent on the secure channel.
2. An adversary can extract all stored data from a lost or stolen smart card based on side channel technology [55, 57, 58].
3. An attacker can easily guess the user identity or password after obtaining information from an intelligent card or public channel according to [56].
4. An adversary can be a legitimate but malicious user or server in the system [4, 33].

## 3. Review of Li et al.’s scheme

This section shows that the scheme proposed by Li et al. [22] has some deficiencies. Li designed three-party password-based authentication key exchange protocol based on chaotic maps providing user anonymity. In his scheme, the information related to user’s password is registered with the server side. Also a modular squaring operation and a square root modulo based on the Chinese Remainder Theorem is used for user anonymity. However, his scheme has some faults in the session key exchange phase and the password change phase. Below is a brief description of the scheme proposed by Li et al. and its deficiencies.

### 3.1 Li et al.’s scheme

#### Notations used in his paper

Table 1 shows some notations used to describe Li et al.’s schemes.

**Table 1:** Notations in Li et al.’s scheme.

Notation	Description
*S* and *A*, *B*	Trusted remote server and two users
*U*_*i*_	Identifier of user *i*
*pw*_*i*_	Password of user *i*
*k*	Secret key of *S*
*p*	A large prime number chosen by *S*
*u*, *v* and *N = u* * *v*	Two large primes maintained by *S*
*p*	A large prime number chosen by *S*
*T*_*n*_(∙)	Chebyshev polynomials of degree *n*
*h*(∙)	One-way hash function
*SQR* (∙), *SQRT* (∙)	Modular squaring operation and square root modulo operation
⊕	XOR operator

#### System initialization

The server selects one private key *k* and two secret large primes (*u*, *v*), and publishes {*p*, *α*, *h*(), *N*}.

#### Registration

Step 1: The user submits {*U*_*i*_, *h* (*rm*_*i*_, *PW*_*i*_)} to *S*, where *rm*_*i*_ ∈ [1, *p*+1] is random number and *PW*_*i*_ = *T*_*pwi*_(*α*).
Step 2: Upon receiving the message from the user, *S* computes *VP*_*i*_ = *h* (*U*_*i*_, *k*) + *h*(*rm*_*i*_, *PW*_*i*_) mod *p* and stores (*U*_*i*_, *VP*_*i*_) in its database.
Step 3: The user stores *rm*_*i*_ into his end-user device.

#### Authentication and key exchange

**Step 1** The user *A* chooses a random number *r*_*A*_ ∈ [1, *p* + 1] and computes *R*_*A*_ = *T*_*rA*_(*α*) mod *p*, *PW*_*A*_ = *T*_*pwA*_(*α*) mod *p* and *h*(*rm*_*A*_, *PW*_*A*_), where *rm*_*A*_ is retrieved from his end-user device. Then *A* sends *M*_1_ = *SQR* (*U*_*A*_, *U*_*B*_, *h*(*rm*_*A*_, *PW*_*A*_), *R*_*A*_) mod *N* to *S*.

**Step 2** Upon receiving *M*_1_ from *A*, *S* obtains (*U*_*A*_, *U*_*B*_, *h*(*rm*_*A*_, *PW*_*A*_)*, *R*_*A*_) = *SQRT*(*M*_1_) by using the Chinese Remainder Theorem with *u* and *v*. Next, *S* retrieves the stored *VP*_*A*_ = *h*(*U*_*A*_, *k*) + *h*(*rm*_*A*_, *PW*_*A*_) corresponding to *U*_*A*_. If *VP*_*A*_—*h*(*U*_*A*_, *k*) = *h*(*rm*_*A*_, *PW*_*A*_)*, then *S* continues next step. *S* chooses two random numbers *r*_*S1*_, *r*_*S2*_∈ [1, *p* + 1] and computes *R*_*S*1_ = *T*_*rS*1_(*α*)- *h*(*rm*_*A*_, *PW*_*A*_) mod *p*, *R*_*S*2_ = *T*_*rS*2_(*α*)—*h*(*rm*_*B*_, *PW*_*B*_) mod *p* and *μ*_*A*_ = *U*_*A*_ ⊕ *T*_*rS*2_(*α*) mod *p*. Then *S* sends *M*_2_ = {*μ*_*A*_, *R*_*A*_, *R*_*S*2_} to *B*.

**Step 3** Upon receiving *M*_2_ from *S*, *B* computes *PW*_*B*_ = *T*_*pwB*_(*α*) mod *p*, *h*(*rm*_*B*_, *PW*_*B*_), *T*_*rS*2_(*α*) = *R*_*S*2_ + *h*(*rm*_*B*_, *PW*_*B*_) and *U*_*A*_ = *μ*_*A*_ ⊕ *T*_*rS*2_(*α*). Then *B* chooses a random number *r*_*B*_ ∈ [1, *p* + 1] and computes *R*_*B*_ = *T*_*rB*_(*α*) mod *p*, *K*_*BS*_ = *T*_*rB*_(*T*_*rS*2_(*α*)) mod *p* = *T*_*rBrS*2_(*α*) mod *p*, *K*_*BA*_ = *T*_*rB*_(*R*_*A*_) mod *p* = *T*_*rBrA*_(*α*) mod *p*, *V*_*BA*_ = *h*(0, *U*_*B*_, *U*_*A*_, *R*_*B*_, *R*_*A*_, *K*_*BA*_) and *V*_*BS*_ = *h*(0, *U*_*B*_, *U*_*A*_, *R*_*B*_, *R*_*S*2_, *V*_*BA*_, *K*_*BS*_). Then *B* sends *M*_3_ = *SQR*(*U*_*B*_, *U*_*A*_, *R*_*B*_, *V*_*BS*_, *V*_*BA*_) mod *N* to *S*.

**Step 4** Upon receiving *M*_3_ from *B*, *S* obtains (*U*_*B*_, *U*_*A*_, *R*_*B*_, *V*_BS_*, *V*_*BA*_) = *SQRT*(*M*_3_). *S* computes *K*_*SB*_ = *T*_*rS*2_(*R*_*B*_) = *T*_*rBrS*2_(*α*) mod *p* and *V*_*BS*_ = *h*(0, *U*_*B*_, *U*_*A*_, *R*_*B*_, *R*_*S*2_, *V*_*BA*_, *K*_*BS*_). If *V*_BS_* = *V*_*BS*_, *B* is authenticated by *S* and then *S* computes *K*_*SA*_ = *T*_*rS*1_(*R*_*A*_) = *T*_*rS*1*rA*_(*α*) mod *p* and *V*_*SA*_ = *h*(0, *U*_*A*_, *U*_*B*_, *R*_*S*1_, *R*_*A*_, *R*_*B*_, *V*_*BA*_, *K*_*SA*_). Then *S* sends *M*_4_ = {*R*_*S*1_, *R*_*B*_, *V*_*BA*_, *V*_*SA*_} to *A*.

**Step A5**: Upon receiving *M*_4_ from *S*, *A* computes *K*_*AS*_ = *T*_*rA*_(*R*_*S*1_ + *h*(*rm*_*A*_, *PW*_*A*_)) = *T*_*rArS*1_(*α*) mod *p* and *V*_*SA*_ = *h*(0, *U*_*A*_, *U*_*B*_, *R*_*S*1_, *R*_*A*_, *R*_*B*_, *V*_*BA*_, *K*_*SA*_). If *V*_*SA*_ equals received *V*_SA_*, *S* is authenticated by *A*. Next, *A* computes *K*_*AB*_ = *T*_*rA*_(*R*_*B*_) mod *p* = *T*_*rArB*_(*α*) mod *p* and *V*_*BA*_ = *h*(0, *U*_*B*_, *U*_*A*_, *R*_*B*_, *R*_*A*_, *K*_*BA*_). If *V*_*BA*_ equals received *V*_BA_*, *B* is authenticated by *A*. *A* computes *V*_*AB*_ = *h*(1, *U*_*A*_, *U*_*B*_, *R*_*A*_, *R*_*B*_, *K*_*AB*_) and *V*_*AS*_ = *h*(1, *U*_*A*_, *U*_*B*_, *R*_*A*_, *R*_*S*1_, *V*_*AB*_, *K*_*AS*_). Then *A* sends *M*_5_ = {*V*_*AS*_, *V*_*AB*_} to *S*.

**Step A6** After receiving *M*_5_ from *A*, *S* verifies if computed *h*(1, *U*_*A*_, *U*_*B*_, *R*_*A*_, *R*_*S*1_, *V*_*AB*_, *K*_*SA*_) equals received *V*_*AS*_. If it holds, *A* is authenticated by *S*. Then *S* computes *V*_*SB*_ = *h*(1, *U*_*A*_, *U*_*B*_, *R*_*A*_, *R*_*B*_, *V*_*AB*_, *K*_*SB*_) and sends *M*_6_ = {*V*_*AB*_, *V*_*SB*_} to *B*.

**Step A7** After receiving *M*_6_ from *S*, *B* verifies if computed *h*(1, *U*_*A*_, *U*_*B*_, *R*_*A*_, *R*_*B*_, *V*_*AB*_, *K*_*SB*_) and *h*(1, *U*_*A*_, *U*_*B*_, *R*_*A*_, *R*_*B*_, *K*_*AB*_) equal received *V*_*SB*_ and *V*_*AB*_. If they are valid, *A* and *S* are authenticated by *B*. Finally, *A* computes the session key *SK*_*AB*_ = *h*(2, *U*_*A*_, *U*_*B*_, *R*_*A*_, *R*_*B*_, *K*_*AB*_) and *B* computes the session key *SK*_*BA*_ = *h*(2, *U*_*A*_, *U*_*B*_, *R*_*A*_, *R*_*B*_, *K*_*AB*_).

#### Password change phase

**Step 1** The user *A* chooses a random number *r*_*A*_ ∈ [1, *p* + 1] and computes *R*_*A*_ = *T*_*rA*_(*α*) mod *p*, *PW*_*A*_ = *T*_*pwA*_(*α*) mod *p* and *h*(*rm*_*A*_, *PW*_*A*_), Then *A* sends *C*_1_ = *SQR*(*U*_*A*_, *h*(*rm*_*A*_, *PW*_*A*_), *R*_*A*_) mod *p* to *S*.

**Step 2** Upon receiving *C*_1_ from *A*, *S* obtains (*U*_*A*_, *h*(*rm*_*A*_, *PW*_*A*_)*, *R*_*A*_) = *SQRT*(C_1_) by using the Chinese Remainder Theorem with *u* and *v*. Next, *S* verifies the received *h*(*rm*_*A*_, *PW*_*A*_)* with the stored *VP*_*A*_ = *h*(*U*_*A*_, *k*)+*h*(*rm*_*A*_, *PW*_*A*_) mod *p* corresponding to *U*_*A*_. If *VP*_*A*_*—h*(*U*_*A*_, *s*) = *h*(*rm*_*A*_, *PW*_*A*_)*, *S* accepts *A*’s request message *C*_1_. Then *S* chooses a random number *r*_*S*_ ∈ [1, *p* + 1] and computes *R*_*S*_ = *T*_*rS*_(*α*)*—h*(*rm*_*A*_, *PW*_*A*_) mod *p*, *K*_*SA*_ = *T*_*rS*_(*R*_*A*_) = *T*_*rSrA*_(*α*) mod *p* and *V*_*SA*_ = *h*(0, *U*_*A*_, *R*_*S*_, *R*_*A*_, *K*_*SA*_). Then *S* sends *C*2 = {*R*_*S*_, *V*_*SA*_} to *A*.

**Step 3** Upon receiving *C*_2_ from *S*, *A* computes *K*_*SA*_ = *T*_*rA*_(*R*_*S*_ + *h*(*rm*_*A*_, *PW*_*A*_)) = *T*_*rSrA*_(*α*) mod *p* and verifies if computed *V*_*SA*_ = *h*(0, *U*_*A*_, *R*_*S*_, *R*_*A*_, *K*_*SA*_) equals received *V*_SA_*. If it holds, *S* is authenticated by *A*. Next, *A* selects a new password *pw*_A_* and a new random number *rm*_A_* and computes *V*_*AS*_ = *h*(1, *U*_*A*_, *R*_*A*_, *R*_*S*_, *K*_*AS*_), *PW*_A_* = *T*_pwA*_ (*α*) mod *p*, and *h*(*rm*_A_*, *PW*_A_*). Then *A* sends *C*_3_ = *SQR*(*V*_*AS*_, *U*_*A*_, *h*(*rm*_A_*, *PW*_A_*)) mod *p* to *S*.

**Step 4** Upon receiving *C*_3_ from *A*, *S* verifies if computed *V*_*AS*_ = *h*(1, *U*_*A*_, *R*_*A*_, *R*_*S*_, *K*_*AS*_) equals received *V*_AS_*. If it holds, *S* accepts *A*’s password change request, computes *R*_1_ = *h*(1, *U*_*A*_, *h*(*rm*_A_*, *PW*_A_*), *K*_*SA*_) and *VP*_A_* = *h*(*U*_*A*_, *k*) + *h*(*rm*_A_*, *PW*_A_*) mod *p* and replaces *VP*_*A*_ with *VP*_A_*. Then *S* sends *C*_4_ = {Accept, *R*_1_} to *A*. Otherwise, *S* rejects *A*’s password change request, computes *R*_2_ = *h*(0, *U*_*A*_, *h*(*rm*_A_*, *PW*_A_*), *K*_*SA*_) and sends *C*_5_ = {Reject, *R*_2_} to *A*. If the message is *C*_4_, *A* verifies if computed *R*_1_ = *h*(1, *U*_*A*_, *h*(*rm*_A_*, *PW*_A_*), *K*_*SA*_) equals received *R*_1_*. If it holds, *A* confirms *pw*_A_* as the new password and replaces *rm*_*A*_ with *rm*_A_* in his end-user device. Otherwise, *A* returns to **Step 1** and follows the process. If the message is *C*_5_, *A* returns to **Step 1** with another new password and follows the process.

### 3.2 Faults of Li et al.’s scheme

Many attack models [54–58] have been proposed by researchers and based on them, cryptographic protocols [25, 33, 34, 38, 59, 60] have been analysed. Based on the adversary model presented in Section 2.6, we analyse Li et al.’ scheme. According to the adversary model, the adversary can eavesdrop and block all message sent on the public channel and he can be a legitimate user in the system. In this paper, we call such an adversary an insider adversary.

#### Verifier disclosure attacks

Li et al.’s scheme has a faults that user’s authentication verifier is disclosed to the insider adversary in the authentication and key exchange phase. In his scheme, *h*(*rm*_*i*_, *PW*_*i*_) is user’s authentication verifier, where *rm*_*i*_ is stored into user’s end-device, *PW*_*i*_ = *T*_*pwi*_(*α*) mod *p* and *pw*_*i*_ is user’s password. However, *h*(*rm*_*i*_, *PW*_*i*_) is disclosed to the insider adversary.

The details of verifier disclosure attack in his scheme are described as follows.

**Step 1**. In order to exchange a session key with a legal user *A*, an inside adversary *C* chooses a random number *r*_*C*_ ∈ [1, *p* + 1] and computes *R*_*C*_ = *T*_*rC*_(*α*) mod *p*, *PW*_*C*_ = *T*_*pwC*_(*α*) mod *p* and *h*(*rm*_*C*_, *PW*_*C*_), where *rm*_*C*_ is retrieved from his end-user device. Then *C* sends *M*_1_ = *SQR* (*U*_*C*_, *U*_*A*_, *h*(*rm*_*C*_, *PW*_*C*_), *R*_*C*_) mod *N* to *S*.

**Step 2**. Upon receiving *M*_1_ from *C*, *S* obtains (*U*_*C*_, *U*_*A*_, *h*(*rm*_*C*_, *PW*_*C*_)*, *R*_*C*_) = *SQRT*(*M*_1_). Next, *S* retrieves the stored *VP*_*C*_ = *h*(*U*_*C*_, *k*) + *h*(*rm*_*C*_, *PW*_*C*_) corresponding to *U*_*C*_. If *VP*_*C*_—*h*(*U*_*C*_, *k*) = *h*(*rm*_*C*_, *PW*_*C*_)*, then *S* continues next step. *S* chooses two random numbers *r*_*S1*_, *r*_*S2*_∈ [1, *p* + 1] and computes *R*_*S*1_ = *T*_*rS*1_(*α*)- *h*(*rm*_*C*_, *PW*_*C*_) mod *p*, *R*_*S*2_ = *T*_*rS*2_(*α*)—*h*(*rm*_*A*_, *PW*_*A*_) mod *p* and *μ*_*C*_ = *U*_*C*_ ⊕ *T*_*rS*2_(*α*) mod *p*. Then *S* sends *M*_2_ = {*μ*_*C*_, *R*_*C*_, *R*_*S*2_} to *A*.

**Step 3**. At this time, *C* intercepts *M*_2_ = {*μ*_C_*, *R*_C_*, *R*_*S*2_*} and computes as follows:

*U*_*C*_ ⊕ *μ*_C_* = *U*_*C*_ ⊕*U*_*C*_ ⊕ *T*_*rS*2_(*α*) *= T*_*rS*2_(*α*) (Because of *U*_*C*_ is *C*’s identifier, *C* knows it. Through checking for *R*_C_* of *M*_2_, *C* can verify that *M*_2_ is a message generated at the server according to *M*_1_.)

$$h\left(rm_{A},PW_{A}\right)=T_{rS}{}_{2}\left(\alpha \right)—R_{S}{}_{2}*=T_{rS}{}_{2}\left(\alpha \right)–\left(T_{rS}{}_{2}\left(\alpha \right)—h\left(rm_{A},PW_{A}\right)\right)$$

As the result, *C* can obtain *A*’s authentication verifier *h*(*rm*_*A*_, *PW*_*A*_).

In this way, *C* can obtain all of legal users’ authentication verifier.

If an insider adversary wants to get an authentication verifier of user *A*, it is necessary to generate a message *M*_1_ for exchanging session key with user *A* according to the designed protocol, send it to the server, intercept the message *M*_2_ from the server, and then compute it according to the procedure shown above.

#### User impersonate attacks

As shown above, since an insider adversary *C* can obtain any of legal users’ authentication verifier through verifier disclosure attack, he can impersonate as any legal user.

If an insider adversary *C* wants to impersonate as a legal user *A* and communicate with *B*, he obtains the user *A*’s authentication verifier *V*_*A*_ = *h*(*rm*_*A*_, *PW*_*A*_) through the verifier disclosure attack as shown above before the authentication and key exchange phases.

In the authentication and key exchange phase, *C* works as follows

**Step 1**. *C* chooses a random number *r*_*C*_ ∈ [1, *p* + 1] and computes *R*_*C*_ = *T*_*rC*_(*α*) mod *p*. After that, he can sufficiently make the message *M*_1_ = *SQR*(*U*_*A*_, *U*_*B*_, *h*(*rm*_*A*_, *PW*_*A*_), *R*_*C*_) mod *N* by using the user *A*’s authentication verifier *V*_*A*_ = *h*(*rm*_*A*_, *PW*_*A*_). Then *C* impersonates *A* to sends *M*_1_ to *S*.

The process in steps 2, 3 and 4 is performed according to the protocol, *B* computes *K*_BA_* = *T*_*rB*_(*R*_*C*_) mod *p* = *T*_*rBrC*_(*α*) mod *p* and *V*_BA_* = *h*(0, *U*_*B*_, *U*_*A*_, *R*_*B*_, *R*_*C*_, *K*_BA_*), *S* computes *K*_SA_* = *T*_*rS*1_(*R*_*C*_) = *T*_*rS*1*rC*_(*α*) mod *p* and *V*_SA_* = *h*(0, *U*_*A*_, *U*_*B*_, *R*_*S*1_, *R*_*C*_, *R*_*B*_, *V*_BA_*, *K*_SA_*).

**Step 5**: *C* intercepts *M*_4_ from *S* to *A*, *C* computes *K*_AS_* = *T*_*rC*_(*R*_*S*1_ + *h*(*rm*_*A*_, *PW*_*A*_)) = *T*_*rCrS*1_(*α*) mod *p*, *K*_AB_* = *T*_*rC*_(*R*_*B*_) mod *p* = *T*_*rCrB*_(*α*) mod *p* and *V*_AB_* = *h*(1, *U*_*A*_, *U*_*B*_, *R*_*C*_, *R*_*B*_, *K*_AB_*) and *V*_AS_* = *h*(1, *U*_*A*_, *U*_*B*_, *R*_*C*_, *R*_*S*1_, *V*_AB_*, *K*_AS_*). Then *A* sends *M*_5_ = {*V*_AS_*, *V*_AB_*} to *S*.

The process in steps 6 and 7 is performed according to the protocol, *B* computes *SK*_BA_* = *h*(2, *U*_*A*_, *U*_*B*_, *R*_*C*_, *R*_*B*_, *K*_BA_*) and *C* computes the session key *SK*_AB_* = *h*(2, *U*_*A*_, *U*_*B*_, *R*_*C*_, *R*_*B*_, *K*_AB_*).

As the result, *C* can successfully impersonate as the user *A*.

#### Failure of user anonymity

An insider adversary *C* can obtain all of legal users’ authentication verifier through verifier disclosure attack as shown above. That is, *C* knows the authentication identifier *V*_*i*_ of any user *U*_*i*_. When a legal user *A* exchanges a session key with a legal user *B*, an insider adversary *C* can intercept *M*_2_ = {*μ*_*A*_, *R*_*A*_, *R*_*S*2_} that *S* sends to *B* and then computes as follows: For each authentication verifier *V*_*i*_ of user *U*_*i*_, *C* repeat the following calculation until *U*_A_* and *U*_*i*_ are equal.


$$T_{rS}{}_{2}*(\alpha )=R_{S}{}_{2}+V_{i}=R_{S}{}_{2}+h(rm_{i},PW_{i})=(T_{rS}{}_{2}(\alpha )—h(rm_{B},PW_{B}))+h(rm_{i},PW_{i})$$

$$U_{A}*=\mu_{A}⊕T_{rS}{}_{2}*(\alpha )\;\text{mod}\;p=U_{A}⊕T_{rS}{}_{2}(\alpha )⊕T_{rS}{}_{2}*(\alpha )$$

If *U*_A_* and *U*_*i*_ are equal, *C* can know that current user’s identifier is *U*_*i*_.

As the result, *C* can know user *A*’s identifier *U*_*A*_.

#### Weaknesses of password change phase

In the Li et al. ‘s scheme, the information related to the user’s password is registered with the remote server and users can change their password in the password change phase. In the registration phase, the information related to the user *U*_*i*_’s password stored on the server is *VP*_*i*_ = *h*(*U*_*i*_, *k*)+*h*(*rm*_*i*_, *PW*_*i*_) (where *h*(*rm*_*i*_, *PW*_*i*_) is user authentication verifier) and this information is replaced with VP*_i_ = *h*(*U*_*i*_, *k*)+*h*(rm*_i_, PW*_i_) in the password change phase. However, an attacker can block the message *C*_3_(user’s request) and *C*_4_ or *C*_5_(server’s response) in Step 4 of the password change phase, then the user cannot know whether his password is successfully changed or not. In this case, if the scheme decides that does not change user’s password, the attacker blocks the message *C*_4_ or *C*_5_, if the scheme decides that changes user’s password, the attacker blocks the message *C*_3_. As the result, the user’s authentication verifier is different with the server’s one, the user cannot login to the server no more.

## 4. Proposed scheme

This section describes an enhanced 3PAKE protocol using smart card that overcomes the limitations of the Li et al.’s scheme. The proposed scheme has five phases: system initialization phase, registration phase, authentication and session key exchange phase, password change phase, and renew registration phase. Table 2 shows some notations used to describe the proposed schemes.

**Table 2:** Notations in the proposed scheme.

Notation	Description
*S* and *A*, *B*	Trusted remote server and two users
*SC*_*i*_	Smart card of user *i*
*U*_*i*_	Identifier of user *i*
*pw*_*i*_	Password of user *i*
*bm*_*i*_	Biometrics of user *i*
*k*_*s*_, *P*_*s*_	Private and public key of *S* (*P*_*s*_ = *T*_*ks*_(*α*))
*N*_*a*_, *N*_*b*_, *N*_*s*_	Random numbers chosen by *A*, *B* and *S*
*p*	A large prime number chosen by *S*
*T*_*n*_(*α*)	Chebyshev polynomials of degree *n*
*H*(∙)	One-way hash function (0,1)* → (0, 1)^n^
*h*(∙)	Bio-hashing function
*E*_*K*_(∙), *D*_*K*_(∙)	Symmetric encrypt and decrypt algorithm with secret key *K*
||	String concatenation operator
⊕	XOR operator

### 4.1 System initialization phase

1. *S* selects his secret key *k*_*s*_ ∈ [1, *p*+1] and computes public key *P*_*s*_ = *T*_*ks*_(*α*).
2. *S* selects a large prime number *p*, *α*∈*Z*_*p*_, *H*(∙) and *E*_*K*_(∙)/*D*_*K*_(∙).

*S* publishes {*p*, *α*, *P*_*s*_, *T*_*n*_(∙), *H*(∙), *E*_*K*_(∙), *D*_*K*_(∙)} as system’s parameters.

### 4.2 User registration phase

**Fig 1:**
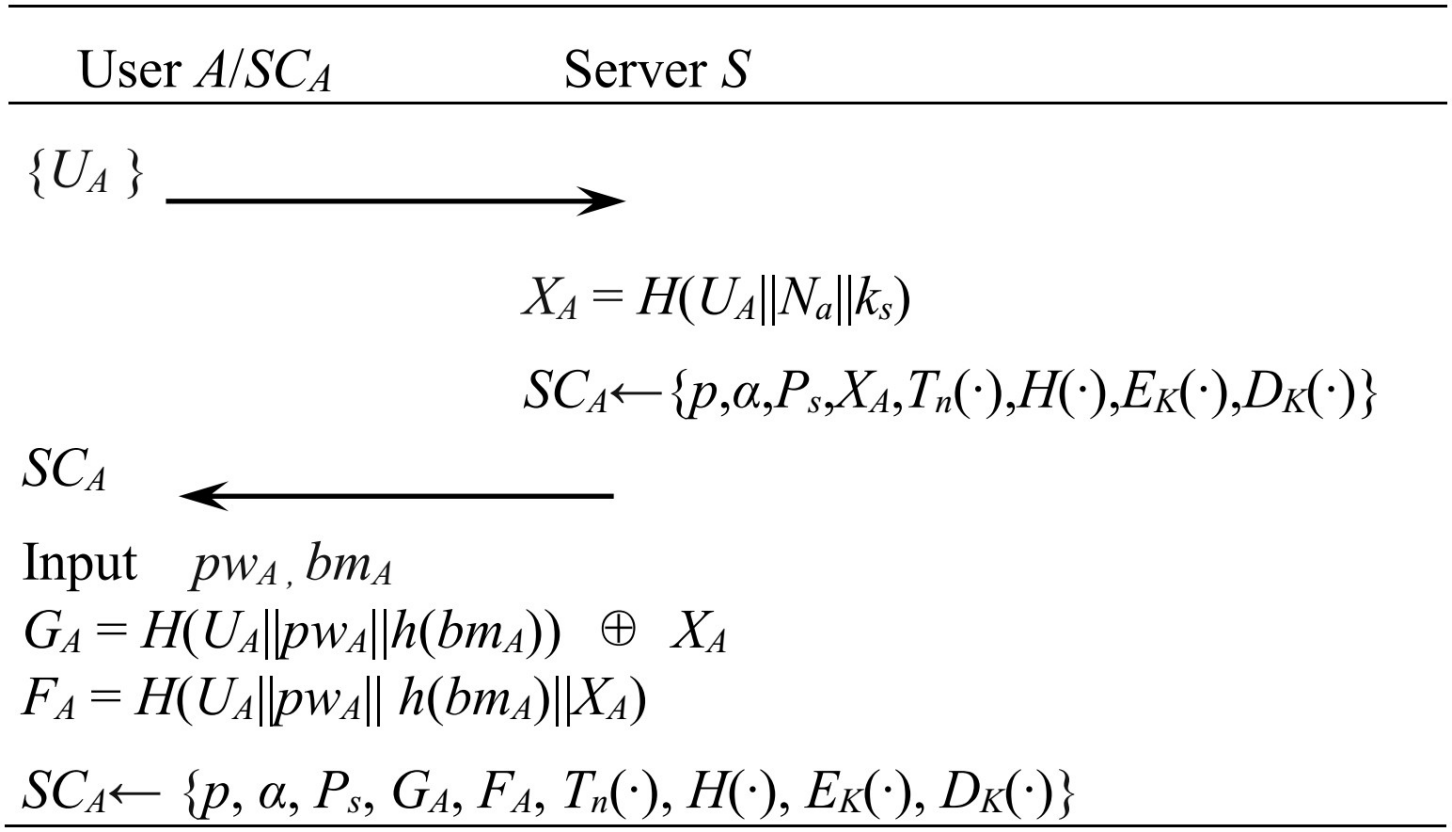
User registration phase.

Fig 1 shows user registration process.

User *A* sends his identifier *U*_*A*_ to *S* via secure channel. *S* retrieves *U*_*A*_ in the user registration table to check whether user *A* has already been registered. If *U*_*A*_ does not exist in the user registration table, *S* chooses a random number *N*_*a*_, computes *X*_*A*_ = *H*(*U*_*A*_||*N*_*a*_||*k*_*s*_) and stores {*p*, *α*, *P*_*s*_, *X*_*A*_, *T*_*n*_(∙), *H*(∙), *E*_*K*_(∙), *D*_*K*_(∙)} in *SC*_*A*_ and delivers it to user *A* via secure channel. *S* stores a tuple {*U*_*A*_, *N*_*a*_} into his user-register table.

User *A*, which receives *SC*_*A*_ from *S*, inputs password *pw*_*A*_ and biometric *bm*_*A*_. The *SC*_*A*_ that receives the user input and computes *G*_*A*_ = *H*(*U*_*A*_||*pw*_*A*_||*h*(*bm*_*A*_)) ⊕ *X*_*A*_, *F*_*A*_ = *H*(*U*_*A*_||*pw*_*A*_|| *h*(*bm*_*A*_)||*X*_*A*_) and stores {*p*, *α*, *P*_*s*_, *G*_*A*_, *F*_*A*_, *T*_*n*_(∙), *H*(∙), *E*_*K*_(∙), *D*_*K*_(∙)} in his memory.

### 4.3 Authentication and session key exchange phase

**Fig 2:**
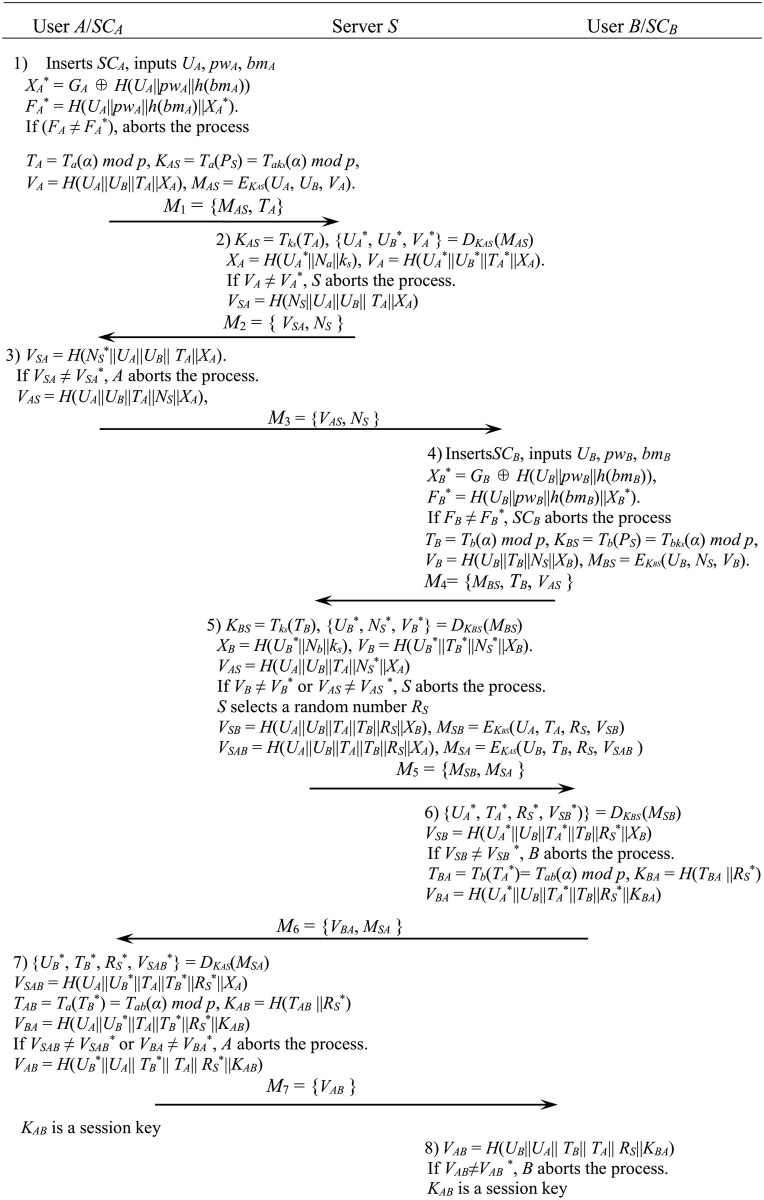
Authentication and session key exchange phase of the proposed scheme.

Fig 2 shows the authentication and session key exchange steps of the proposed scheme.

**Step 1**. User *A* connects his smart card *SC*_*A*_ to the user end-device and inputs his identifier *U*_*A*_, password *pw*_*A*_ and biometrics *bm*_*A*_. *SC*_*A*_ computes

$${X_{A}}^{*}=G_{A}⊕H\left(U_{A}∥pw_{A}∥h\left(bm_{A}\right)\right),{F_{A}}^{*}=H\left(U_{A}∥pw_{A}∥h\left(bm_{A}\right)∥{X_{A}}^{*}\right).$$

If *F*_*A*_ ≠ *F*_A_*, *SC*_*A*_ aborts the process. Otherwise *SC*_*A*_ selects any *a*∈ [1, *p*+1] and computes

$$T_{A}=T_{a}(\alpha )\;mod\;p,K_{AS}=T_{a}(P_{S})=T_{aks}(\alpha )\;mod\;p$$

$$V_{A}=H(U_{A}∥U_{B}∥T_{A}∥X_{A}),M_{AS}=E_{KAS}(U_{A},U_{B},V_{A})$$

*A* sends *M*_1_ = {*M*_*AS*_, *T*_*A*_} to *S*.

**Step 2**. After receiving *M*_1_ = {*M*_*AS*_, *T*_*A*_} from *A*, *S* computes

$$K_{AS}=T_{ks}\left(T_{A}\right)=T_{ksa}\left(\alpha \right)\;mod\;p,\left\{U_{A}*,U_{B}*,V_{A}*\right\}\;=D_{KAS}\left(M_{AS}\right),X_{A}=H\left(U_{A}*∥N_{a}∥k_{s}\right),V_{A}=H(U_{A}*∥U_{B}*|T_{A}*|X_{A}).$$

*S* checks whether *V*_*A*_ and *V*_A_* are same. If *V*_*A*_ ≠ *V*_A_*, *S* aborts the process. *S* chooses a random number *Ns* ∈ [1, *p*+1] and computes *V*_*SA*_ = *H*(*N*_*S*_||*U*_*A*_||*U*_*B*_|| *T*_*A*_||*X*_*A*_).

*S* sends *M*_2_ = {*V*_*SA*_, *N*_*S*_} to *A*.

**Step 3**. After receiving *M*_2_ = {*V*_SA_*, *N*_S_*} from *S*, *A* computes *V*_*SA*_ = *H*(*N*_S_*||*U*_*A*_||*U*_*B*_|| *T*_*A*_||*X*_*A*_).

*A* checks whether *V*_*SA*_ and *V*_SA_* are same. If *V*_*SA*_ ≠ *V*_SA_*, *A* aborts the process. *A* computes *V*_*AS*_ = *H*(*U*_*A*_||*U*_*B*_||*T*_*A*_||*N*_*S*_||*X*_*A*_) and sends *M*_3_ = {*V*_*AS*_, *N*_*S*_} to *B*.

**Step 4**. After receiving *M*_3_ = {*V*_AS_*, *N*_S_*} from *A*, *B* connects his smart card *SC*_*B*_ to the user end-device and inputs his identifier *U*_*B*_, password *pw*_*B*_ and biometrics *bm*_*B*_. *SC*_*B*_ computes

$${X_{B}}^{*}=G_{B}⊕H\left(U_{B}∥pw_{B}∥h\left(bm_{B}\right)\right),{F_{B}}^{*}=H\left(U_{B}∥pw_{B}∥h\left(bm_{B}\right)∥{X_{B}}^{*}\right).$$

If *F*_*B*_ ≠ *F*_B_*, *SC*_*B*_ aborts the process. Otherwise *SC*_*B*_ computes

$$T_{B}=T_{b}(\alpha )\;mod\;p,K_{BS}=T_{b}(P_{S})=T_{bks}(\alpha )\;mod\;p$$

$$V_{B}=H(U_{B}∥T_{B}∥N_{S}∥X_{B}),M_{BS}=E_{KBS}(U_{B},N_{S},V_{B})$$

*B* sends *M*_4_ = {*M*_*BS*_, *T*_*B*_, *V*_*AS*_} to *S*.

**Step 5**. After receiving *M*_4_ = {*M*_*BS*_, *T*_B_*, *V*_AS_*} from *B*, *S* computes

$$K_{BS}=T_{ks}(T_{B})=T_{bks}(\alpha )\;mod\;p,\{{U_{B}}^{*},{N_{S}}^{*},{V_{B}}^{*}\}\;=D_{KBS}(M_{BS}),$$

$$X_{B}=H\left({U_{B}}^{*}∥N_{b}∥k_{s}\right),V_{B}=H\left({U_{B}}^{*}∥{T_{B}}^{*}∥{N_{S}}^{*}∥X_{B}\right),V_{AS}=H\left(U_{A}∥U_{B}∥T_{A}∥{N_{S}}^{*}∥X_{A}\right)$$

If *V*_*B*_ ≠ *V*_B_* or *V*_*AS*_ ≠ *V*_*AS*_ *, *S* aborts the process. Otherwise *S* authenticates *A* and *B*, and chooses a random number *R*_*S*_, then computes

$$V_{SB}=H(U_{A}∥U_{B}∥T_{A}∥T_{B}∥R_{S}∥X_{B}),M_{SB}=E_{KBS}(U_{A},T_{A},R_{S},V_{SB}),$$

$$V_{SAB}=H(U_{A}∥U_{B}∥T_{A}∥T_{B}∥R_{S}∥X_{A}),M_{SA}=E_{KAS}(U_{B},T_{B},R_{S},V_{SAB})$$

*S* sends *M*_5_ = {*M*_*SB*_, *M*_*SA*_} to *B*.

**Step 6**. After receiving *M*_5_ = {*M*_*SB*_, *M*_*SA*_} from *S*, *B* computes

$$\left\{{U_{A}}^{*},{T_{A}}^{*},{R_{S}}^{*},V_{SB}^{*})\right\}=D_{KBS}\left(M_{SB}\right),\;V_{SB}=H\left({U_{A}}^{*}∥U_{B}∥{T_{A}}^{*}∥T_{B}∥{R_{S}}^{*}∥X_{B}\right)$$

If *V*_*SB*_ ≠ *V*_SB_*, *B* aborts the process. Otherwise *B* authenticates *S* and *A*, and then computes

$$T_{BA}=T_{b}({T_{A}}^{*})=T_{ab}(\alpha )\;mod\;p,K_{BA}=H(T_{BA}∥R_{S})$$

$$V_{BA}=H\left({U_{A}}^{*}∥U_{B}∥{T_{A}}^{*}∥T_{B}∥{R_{S}}^{*}∥K_{BA}\right)$$

*B* sends *M*_6_ = {*V*_*BA*_, *M*_*SA*_} to *A*.

**Step 7**. After receiving *M*_6_ = {*V*_BA_*, *M*_*SA*_} from *B*, *A* computes

$$\left\{{U_{B}}^{*},{T_{B}}^{*},{R_{S}}^{*},V_{SAB}^{*}\right\}=D_{KAS}\left(M_{SA}\right),V_{SAB}=H\left(U_{A}∥{U_{B}}^{*}∥T_{A}∥{T_{B}}^{*}∥{R_{S}}^{*}|X_{A}\right)$$

$$T_{AB}=T_{a}({T_{B}}^{*})=T_{ab}(\alpha )\;mod\;p,K_{AB}=H(T_{AB}∥R_{S})$$

$$V_{BA}=H\left(U_{A}∥{U_{B}}^{*}∥T_{A}∥{T_{B}}^{*}∥{R_{S}}^{*}∥K_{AB}\right)$$

If *V*_*SAB*_ ≠ *V*_SAB_* or *V*_*BA*_ ≠ *V*_BA_*, *A* aborts the process. Otherwise *A* authenticates *B* and *S*. *A* sets *K*_*AB*_ as a session key. Then *A* computes *V*_*AB*_ = *H*(*U*_B_*|| *U*_*A*_|| *T*_B_*|| *T*_*A*_||*R*_S_*||*K*_*AB*_)

*A* sends *M*_7_ = {*V*_*AB*_} to *B*.

**Step 8**. After receiving *M*_7_ = {*V*_AB_*} from *A*, *B* computes

$$V_{AB}=H(U_{B}∥U_{A}∥T_{B}∥T_{A}∥R_{S}∥K_{AB})$$

If *V*_*AB*_≠ *V*_AB_*, *B* aborts the process. Otherwise *B* authenticates *A* and sets *K*_*BA*_ as a session key.

### 4.4 Password change phase

User *A* connects his smart card *SC*_*A*_ to the user end-device and inputs his identifier *U*_*A*_, password and biometrics *bm*_*A*_. *SC*_*A*_ computes *X*_A_* = *G*_*A*_ ⊕ *H*(*U*_*A*_||*pw*_*A*_||*h*(*bm*_*A*_)) and *F*_A_* = *H*(*U*_*A*_||*pw*_*A*_||*h*(*bm*_*A*_)||*X*_A_*), and checks whether *F*_*A*_ and *F*_A_* are same. If *F*_*A*_ ≠ *F*_A_*, *SC*_*A*_ aborts the process. Otherwise *SC*_*A*_ requests the user to input a new password *npw*_*A*_. *SC*_*A*_ computes *G*_*A*_^*new*^ = *H*(*U*_*A*_||*npw*_*A*_||*h*(*bm*_*A*_)) ⊕ *X*_*A*_, *F*_*A*_^*new*^ = *H*(*U*_*A*_||*npw*_*A*_|| *h*(*bm*_*A*_)||*X*_*A*_) and replaces <*G*_*A*_, *F*_*A*_> of his memory with <*G*_*A*_^*new*^, *F*_*A*_^*new*^>.

### 4.5 Re-registration phase

When a user registered with the server has lost or stolen his smart card, he needs to re-register with the server. But, some schemes [19, 22, 23] have not the re-registration phase or cannot re-register without changing his identifier, because the user’s secret consists of user’s identifier and server’s secret.

In the proposed scheme, as the user’s secret *X*_*A*_ consists of user’s identifier, random number and server’s secret, users can re-register with the remote server without changing his identifier. If a user wants to re-register with the server, he should only send his identifier to the server and register with the server following the proposed registration phase scheme.

User *A* sends his identifier *U*_*A*_ to *S* via secure channel. *S* retrieves *U*_*A*_ in the user registration table to check whether user *A* has already been registered. If *U*_*A*_ exists in the user registration table, *S* chooses a random number *N*_*a*_^*new*^, computes *X*_*A*_^*new*^ = *H*(*U*_*A*_||*N*_*a*_^*new*^||*k*_*s*_) and stores {*p*, *α*, *P*_*s*_, *X*_*A*_^*new*^, *T*_*n*_(∙), *H*(∙), *E*_*K*_(∙), *D*_*K*_(∙)} in *SC*_*A*_ and delivers it to user *A* via secure channel. *S* stores a tuple {*U*_*A*_, *N*_*a*_^*new*^} into his user-register table.

User *A*, which receives *SC*_*A*_ from *S*, inputs password *pw*_*A*_ and biometric *bm*_*A*_. The *SC*_*A*_ that receives the user input and computes *G*_*A*_ = *H*(*U*_*A*_||*pw*_*A*_||*h*(*bm*_*A*_)) ⊕ *X*_*A*_^*new*^, *F*_*A*_ = *H*(*U*_*A*_||*pw*_*A*_|| *h*(*bm*_*A*_)||*X*_*A*_^*new*^) and stores {*p*, *α*, *P*_*s*_, *GX*_*A*_, *GK*_*A*_, *F*_*A*_, *T*_*n*_(∙), *H*(∙), *E*_*K*_(∙), *D*_*K*_(∙)} in his memory.

## 5. Security analysis of the proposed scheme

In this section, we present an informal analysis and formal verification of the proposed scheme.

For formal analysis, we first use the BAN logic [49] to verify the mutual authentication property and the validation of the established session key of the proposed scheme, and we next use AVISPA (Automated validation of internet security protocol and application) toolkit [50] to verify the resistance of the proposed scheme against the passive and active attacks including man-in-the-middle and replay attacks.

Last, we demonstrate the proposed scheme can resist various kinds of attacks and provides various security properties through informal security analysis.

### 5.1 Authentication proof based on BAN logic

#### Notations and rules

Table 3 shows some notations and rules defined in BAN logic [49].

**Table 3:** Shows some notations and rules of BAN logic.

Notation	Description
*P* |≡ *X*	*P* believes *X*
*P* ⊲ *X*	*P* sees *X*
*P* |∼ *X*	*P* once said *X*
*P* |⇒ *X*	*P* has jurisdiction over *X*
#(*X*)	*X* is fresh
$P\overset{K}{↔}Q$	*K* is a shared secret key between *P* and *Q*
{*X*}_*K*_	Formula *X* are encrypted under the key *K*
<*X*>_*Y*_	*X* combined with the formula *Y*
$R_{1}:\frac{P|\equiv Q\overset{K}{\Leftrightarrow }P,\;P⊲\;<X{>}_{K}}{P|\equiv \;Q|\sim X},\frac{P|\equiv Q\overset{K}{↔}P,\;P⊲{\left\{X\right\}}_{K}}{P|\equiv \;Q|\sim X}$	Message-meaning rule
$R_{2}:\frac{P|\equiv \#(X),\;P|\equiv \;Q|\sim X}{P|\equiv \;Q|\equiv X}$	Nonce-verification rule
$R_{3}:\frac{P|\equiv Q|\Rightarrow X,\;P|\equiv \;Q|\equiv X}{P|\equiv \;X}$	Jurisdiction rule
$R_{4}:\frac{P|\equiv \#(X)}{P|\equiv \;\#(X,Y)}$	Freshness rule
$R_{5}:\frac{P|\equiv Q|\equiv (X,\;Y)}{P|\equiv \;Q|\equiv X},\frac{P|\equiv X,\;P|\equiv Y}{P|\equiv \;(X,Y)}$	Belief rule
$R_{7}:\frac{P|\equiv Q\overset{K}{↔}P,\;P⊲{\left\{X\right\}}_{K}}{P⊲\;X}$	See rule
$R_{8}:\frac{P|\equiv Q|\sim H(X),\;P⊲X}{P|\equiv Q|\sim X}$	Hash function rule

#### Goals

We establish the following goals to prove that our scheme provides strong mutual authentication and the established session key is secure.

*Goal*_1_:*S*|≡*A*|≡*U*_*A*_
*Goal*_2_:*S*|≡*B*|≡*U*_*B*_
*Goal*_3_:*A*|≡*S*|≡*R*_*S*_
*Goal*_4_:*B*|≡*S*|≡*R*_*S*_
*Goal*_5_:*A*|≡*B*|≡*U*_*B*_
*Goal*_6_:*B*|≡*U*_*A*_

$Goal_{7}:\;A|\equiv \;A\overset{K_{AB}}{↔}B$

$Goal_{8}:\;B|\equiv \;A\overset{K_{AB}}{↔}B$

$Goal_{9}:\;A|\equiv \;B|\equiv A\overset{K_{AB}}{↔}B$

$Goal_{10}:\;B|\equiv \;A|\equiv A\overset{K_{AB}}{↔}B$

#### Idealize

We idealize the messages of the proposed scheme as follows:

$M_{AS}:A\to S:{\left\{U_{A},\;U_{B},\;V_{A}=<H(U_{A}∥U_{B}∥T_{A}∥A\overset{X_{A}}{↔}S){>}_{A\overset{X_{A}}{↔}S}\right\}}_{K_{AS}},\;T_{A}$

$M_{2}:S\to A:V_{SA}=<H(N_{S}∥U_{A}∥U_{B}∥T_{A}∥A\overset{X_{A}}{↔}S){>}_{A\overset{X_{A}}{↔}S},\;N_{S}$

$M_{3}:A\to S:V_{AS}=<H(U_{A}∥U_{B}∥T_{A}∥N_{S}∥A\overset{X_{A}}{↔}S){>}_{A\overset{X_{A}}{↔}S},N_{S}$

$M_{BS}:B\to S:{\left\{U_{B},N_{S},\;V_{B}=<H(U_{B}∥T_{B}∥N_{S}∥B\overset{X_{B}}{↔}S){>}_{B\overset{X_{B}}{↔}S}\right\}}_{K_{BS}},\;T_{B}$

$M_{SB}:S\to B:{\left\{U_{A},T_{A},\;V_{SB}=<H(U_{A}∥U_{B}∥T_{A}∥T_{B}∥R_{S}∥B\overset{X_{B}}{↔}S){>}_{B\overset{X_{B}}{↔}S}\right\}}_{K_{BS}}$

$M_{SA}:S\to A:{\left\{U_{B},T_{B},\;V_{SAB}=<H(U_{A}∥U_{B}∥T_{A}∥T_{B}∥R_{S}∥A\overset{X_{A}}{↔}S){>}_{A\overset{X_{A}}{↔}S}\right\}}_{K_{AS}}$

*M*_6_:*B* → *A*:*V*_*BA*_ = *H*(*U*_*A*_‖*U*_*B*_‖*T*_*A*_‖*T*_*B*_‖*R*_*S*_‖*K*_*AB*_)
*M*_7_:*A* → *B*:*V*_*AB*_ = *H*(*U*_*B*_‖*U*_*A*_‖*T*_*B*_‖*T*_*A*_‖*R*_*S*_‖*K*_*AB*_)

#### Assumptions

The initial assumptions of the proposed scheme are as follows:

*A*_*A*1_: *A*|≡*a*
*A*_*A*2_: *A*|≡#(*a*)
*A*_A3_: *A*|≡*K*_*AS*_

$A_{A4}:A|\equiv A\overset{X_{A}}{↔}S$

*A*_*A*5_: *A*|≡*U*_*A*_
*A*_*A*6_: *A*|≡*U*_*B*_
*A*_*A*7_: *A*|≡*S*|⇒*T*_*B*_
*A*_*A*8_: *A*|≡*S*|⇒*R*_*S*_
*A*_*B*1_: *B*|≡*b*
*A*_*B*2_: *B*|≡#(*b*)
*A*_B3_: *B*|≡*K*_*BS*_

$A_{B4}:B|\equiv B\overset{X_{B}}{↔}S$

*A*_*B*5_: *B*|≡*U*_*B*_
*A*_*B*6_: *B*|≡*S*|⇒*T*_*A*_
*A*_*B*7_: *B*|≡*S*|⇒*R*_*S*_
*A*_*S*1_: *S*|≡*N*_*S*_
*A*_*S*2_: *S*|≡#(*N*_*S*_)

$A_{S3}:S|\equiv A\overset{X_{A}}{↔}S$

$A_{S4}:S|\equiv B\overset{X_{B}}{↔}S$

#### Analysis

According to *M*_*AS*_ and *A*_*S*3_, we apply the Message-meaning rule (*R*_1_) and Hash function rule(*R*_8_), we can obtain:

$$\begin{array}{ll} S_{1}: & \frac{S⊲K_{AS},\;S⊲{\left\{U_{A},\;U_{B},\;V_{A}=<H(U_{A}∥U_{B}∥T_{A}∥A\overset{X_{A}}{↔}S){>}_{A\overset{X_{A}}{↔}S}\right\}}_{K_{AS}}}{S⊲\{U_{A},\;U_{B},\;<H(U_{A}∥U_{B}∥T_{A}∥A\overset{X_{A}}{↔}S){>}_{A\overset{X_{A}}{↔}S}\},\;S⊲T_{A}},\\ & \frac{S|\equiv A\overset{X_{A}}{↔}S,\;S⊲<H(U_{A}∥U_{B}∥T_{A}∥A\overset{X_{A}}{↔}S){>}_{A\overset{X_{A}}{↔}S}}{S|\equiv \;A|\sim H(U_{A}∥U_{B}∥T_{A}∥A\overset{X_{A}}{↔}S)},\\ & \frac{S|\equiv \;A|\sim H(U_{A}∥U_{B}∥T_{A}∥A\overset{X_{A}}{↔}S),S⊲\{U_{A},U_{B},T_{A}\}}{S|\equiv \;A|\sim (U_{A},U_{B},T_{A})} \end{array}\;\;\;\;\;\;$$

According to *M*_2_ and *A*_*A*4_, we apply the Message-meaning rule (*R*_1_) and Hash function rule(*R*_8_), we can obtain:

$$\begin{array}{ll} S_{2}: & \frac{A|\equiv A\overset{X_{A}}{↔}S,\;A⊲<H(N_{S}∥U_{A}∥U_{B}∥T_{A}∥A\overset{X_{A}}{↔}S){>}_{A\overset{X_{A}}{↔}S}}{A|\equiv \;S|\sim H(N_{S}∥U_{A}∥U_{B}∥T_{A}∥A\overset{X_{A}}{↔}S)},\\ & \frac{A|\equiv S|\sim H(N_{S}∥U_{A}∥U_{B}∥T_{A}∥A\overset{X_{A}}{↔}S),A⊲\{N_{S},U_{A},U_{B},T_{A}\}}{A|\equiv S|\sim (N_{S},U_{A},U_{B},T_{A})} \end{array}\;\;\;\;\;\;$$

According to *M*_2_, Freshness rule(*R*_4_) and *A*_*A*2_, we can obtain:

$$\begin{array}{ll} S_{3}: & \frac{A|\equiv \#(a),T_{A}=T_{a}(\alpha )}{A|\equiv \#(T_{A})},\frac{A|\equiv \#(T_{A})}{A|\equiv \#H(N_{S}∥U_{A}∥U_{B}∥T_{A}∥A\overset{X_{A}}{↔}S)},\\ & \frac{A|\equiv \#H(N_{S}∥U_{A}∥U_{B}∥T_{A}∥A\overset{X_{A}}{↔}S)}{A|\equiv \#(N_{S},U_{A},U_{B},T_{A})}, \end{array}\;\;\;\;$$

According to *S*_2_ and *S*_3_, we apply the Nonce-verification rule (*R*_2_) and Belief rule(*R*_5_), we can obtain:

$$\begin{array}{ll} S_{4}: & \frac{A|\equiv \#(N_{S},U_{A},U_{B},T_{A}),A|\equiv S|\sim (N_{S},U_{A},U_{B},T_{A})}{A|\equiv S|\equiv (N_{S},U_{A},U_{B},T_{A})}\\ & \frac{A|\equiv S|\equiv (N_{S},U_{A},U_{B},T_{A})}{A|\equiv S|\equiv N_{S}} \end{array}\;\;\;\;\;$$

According to *M*_3_, *A*_*S*3_ and *S*_1_, we apply the Message-meaning rule (*R*_1_) and Hash function rule(*R*_8_), we can obtain:

$$\begin{array}{ll} S_{5}: & \frac{S|\equiv A\overset{X_{A}}{↔}S,\;S⊲<H(U_{A}∥U_{B}∥T_{A}∥N_{S}∥A\overset{X_{A}}{↔}S){>}_{A\overset{X_{A}}{↔}S}}{S|\equiv A|\sim H(U_{A}∥U_{B}∥T_{A}∥N_{S}∥A\overset{X_{A}}{↔}S)},\\ & \frac{S|\equiv A|\sim H(U_{A}∥U_{B}∥T_{A}∥N_{S}∥A\overset{X_{A}}{↔}S),S⊲\{N_{S},U_{A},U_{B},T_{A}\}}{S|\equiv A|\sim (N_{S},U_{A},U_{B},T_{A})} \end{array}\;\;\;\;\;$$

According to *M*_3_, Freshness rule(*R*_4_) and *A*_*S*2_, we can obtain:

$$\begin{array}{ll} S_{6}: & \frac{S|\equiv \#(N_{S})}{S|\equiv \#H(N_{S}∥U_{A}∥U_{B}∥T_{A}∥A\overset{X_{A}}{↔}S)},\\ & \frac{S|\equiv \#H(N_{S}∥U_{A}∥U_{B}∥T_{A}∥A\overset{X_{A}}{↔}S)}{S|\equiv \#(N_{S},U_{A},U_{B},T_{A})}, \end{array}\;\;\;\;\;\;$$

According to *S*_5_ and *S*_6_, we apply the Nonce-verification rule (*R*_2_) and Belief rule(*R*_5_), we can obtain:

$$\begin{array}{ll} S_{7}: & \frac{S|\equiv \#(N_{S},U_{A},U_{B},T_{A}),S|\equiv A|\sim (N_{S},U_{A},U_{B},T_{A})}{S|\equiv A|\equiv (N_{S},U_{A},U_{B},T_{A})},\\ & \;\frac{S|\equiv A|\equiv (N_{S},U_{A},U_{B},T_{A})}{S|\equiv A|\equiv T_{A},S|\equiv A|\equiv U_{A}} \end{array}:Goal_{1}$$

According to *M*_*BS*_ and *A*_*S*4_, we apply the Message-meaning rule (*R*_1_) and Hash function rule(*R*_8_), we can obtain:

$$\begin{array}{l} \begin{array}{ll} S_{8}: & \frac{S⊲K_{BS},\;S⊲{\left\{U_{B},N_{S},V_{B}=<H(U_{B}∥T_{B}∥N_{S}∥B\overset{X_{B}}{↔}S){>}_{B\overset{X_{B}}{↔}S}\right\}}_{K_{BS}}}{S⊲\{U_{B},N_{S},<H(U_{B}∥T_{B}∥N_{S}∥B\overset{X_{B}}{↔}S){>}_{B\overset{X_{B}}{↔}S}\},S⊲T_{B}},\\ & \frac{S|\equiv B\overset{X_{B}}{↔}S,S⊲<H(U_{B}∥T_{B}∥N_{S}∥B\overset{X_{B}}{↔}S){>}_{B\overset{X_{B}}{↔}S}}{S|\equiv B|\sim H(U_{B}∥T_{B}∥N_{S}∥B\overset{X_{B}}{↔}S)},\\ & \frac{S|\equiv B|\sim H(U_{B}∥T_{B}∥N_{S}∥B\overset{X_{B}}{↔}S),S⊲\{U_{B},T_{B},N_{S}\}}{S|\equiv B|\sim (U_{B},T_{B},N_{S})} \end{array}\;\;\;\;\\ \;\;\;\;\;\;\;\; \end{array}$$

According to *M*_*BS*_, Freshness rule(*R*_4_) and *A*_*S*2_, we can obtain:

$$\begin{array}{ll} S_{9}: & \frac{S|\equiv \#(N_{S})}{S|\equiv \#H(U_{B}∥T_{B}∥N_{S}∥B\overset{X_{B}}{↔}S)},\\ & \frac{S|\equiv \#H(U_{B}∥T_{B}∥N_{S}∥B\overset{X_{B}}{↔}S)}{S|\equiv \#(U_{B},T_{B},N_{S})} \end{array}\;\;\;\;\;$$

According to *S*_8_ and *S*_9_, we apply the Nonce-verification rule (*R*_2_) and Belief rule(*R*_5_), we can obtain:

$$\begin{array}{ll} S_{10}: & \frac{S|\equiv \#(U_{B},T_{B},N_{S}),S|\equiv B|\sim (U_{B},T_{B},N_{S})}{S|\equiv B|\equiv (U_{B},T_{B},N_{S})},\\ & \frac{S|\equiv B|\equiv (U_{B},T_{B},N_{S})}{S|\equiv B|\equiv T_{B},S|\equiv B|\equiv U_{B}} \end{array}\;\;\;\;\;\;:Goal_{2}$$

According to *M*_*SB*_ and *A*_*B*4_, we apply the Message-meaning rule (*R*_1_) and Hash function rule(*R*_8_), we can obtain:

$$\begin{array}{l} \begin{array}{ll} S_{11}: & \frac{B⊲K_{BS},B⊲{\left\{U_{A},T_{A},R_{S},<H(U_{A}∥U_{B}∥T_{A}∥T_{B}∥R_{S}∥B\overset{X_{B}}{↔}S){>}_{B\overset{X_{B}}{↔}S}\right\}}_{K_{BS}}}{B⊲\{U_{A},T_{A},R_{S},<H(U_{A}∥U_{B}∥T_{A}∥T_{B}∥R_{S}∥B\overset{X_{B}}{↔}S){>}_{B\overset{X_{B}}{↔}S}\}},\\ & \frac{B|\equiv B\overset{X_{B}}{↔}S,B⊲<H(U_{A}∥U_{B}∥T_{A}∥T_{B}∥R_{S}∥B\overset{X_{B}}{↔}S){>}_{B\overset{X_{B}}{↔}S}}{B|\equiv S|\sim H(U_{A}∥U_{B}∥T_{A}∥T_{B}∥R_{S}∥B\overset{X_{B}}{↔}S)},\\ & \frac{B|\equiv S|\sim H(U_{A}∥U_{B}∥T_{A}∥T_{B}∥R_{S}∥B\overset{X_{B}}{↔}S),B⊲\{U_{A},U_{B},T_{A},T_{B},R_{S}\}}{B|\equiv S|\sim (U_{A},U_{B},T_{A},T_{B},R_{S})} \end{array}\;\;\;\;\\ \;\;\;\;\;\;\;\; \end{array}$$

According to *M*_*SB*_, Freshness rule(*R*_4_) and *A*_*B*2_, we can obtain:

$$\begin{array}{ll} S_{12}: & \frac{B|\equiv \#(b),T_{B}=T_{b}(\alpha )}{B|\equiv \#(T_{B})},\frac{B|\equiv \#(T_{B})}{B|\equiv \#H(U_{A}∥U_{B}∥T_{A}∥T_{B}∥R_{S}∥B\overset{X_{B}}{↔}S)},\\ & \frac{B|\equiv \#H(U_{A}∥U_{B}∥T_{A}∥T_{B}∥R_{S}∥B\overset{X_{B}}{↔}S)}{B|\equiv \#(U_{A},U_{B},T_{A},T_{B},R_{S})} \end{array}\;\;\;\;\;$$

According to *S*_11_ and *S*_12_, we apply the Nonce-verification rule (*R*_2_) and Belief rule(*R*_5_), we can obtain:

$$\begin{array}{ll} S_{13}: & \frac{B|\equiv \#(U_{A},U_{B},T_{A},T_{B},R_{S}),B|\equiv S|\sim (U_{A},U_{B},T_{A},T_{B},R_{S})}{B|\equiv S|\equiv (U_{A},U_{B},T_{A},T_{B},R_{S})},\\ & \frac{B|\equiv S|\equiv (U_{A},U_{B},T_{A},T_{B},R_{S})}{B|\equiv S|\equiv R_{S},B|\equiv S|\equiv U_{A},B|\equiv S|\equiv T_{A}} \end{array}:Goal_{4}\;\;\;\;\;\;$$

According to *S*_13_ and *A*_*B*6_, we apply the Jurisdiction rule (*R*_3_), we can obtain:

$$S_{14}:\frac{B|\equiv S|\Rightarrow U_{A},B|\equiv S|\equiv U_{A}}{B|\equiv U_{A}}:Goal_{6}$$

According to *S*_13_, *A*_*B*7_ and *A*_*B*8_, we apply the Jurisdiction rule (*R*_3_), we can obtain:

$$S_{15}:\frac{B|\equiv S|\Rightarrow T_{A},B|\equiv S|\equiv T_{A}}{B|\equiv T_{A}},\frac{B|\equiv S|\Rightarrow R_{S},B|\equiv S|\equiv R_{S}}{B|\equiv R_{S}}$$

According to *S*_15_, *A*_*B*1_ and *K*_*AB*_ = *H*(*T*_*AB*_ || *R*_*S*_), we apply the Belief rule(*R*_5_), we can obtain:

$$S_{16}:\frac{B|\equiv T_{A},B|\equiv b}{B|\equiv T_{AB}(=T_{b}(T_{A}))},\frac{B|\equiv T_{AB},B|\equiv R_{S}}{B|\equiv A\overset{K_{AB}}{↔}B}:Goal_{8}$$

According to *M*_*SA*_ and *A*_*A*4_, we apply the Message-meaning rule (*R*_1_) and Hash function rule(*R*_8_), we can obtain:

$$\begin{array}{l} \begin{array}{ll} S_{17}: & \frac{A⊲K_{AS},A⊲{\left\{U_{B},T_{B},R_{S},<H(U_{A}∥U_{B}∥T_{A}∥T_{B}∥R_{S}∥A\overset{X_{A}}{↔}S){>}_{A\overset{X_{A}}{↔}S}\right\}}_{K_{AS}}}{A⊲\{U_{B},T_{B},R_{S},<H(U_{A}∥U_{B}∥T_{A}∥T_{B}∥R_{S}∥A\overset{X_{A}}{↔}S){>}_{A\overset{X_{A}}{↔}S}\}},\\ & \frac{A|\equiv A\overset{X_{A}}{↔}S,A⊲<H(U_{A}∥U_{B}∥T_{A}∥T_{B}∥R_{S}∥A\overset{X_{A}}{↔}S){>}_{A\overset{X_{A}}{↔}S}}{A|\equiv S|\sim H(U_{A}∥U_{B}∥T_{A}∥T_{B}∥R_{S}∥A\overset{X_{A}}{↔}S)},\\ & \frac{A|\equiv S|\sim H(U_{A}∥U_{B}∥T_{A}∥T_{B}∥R_{S}∥A\overset{X_{A}}{↔}S),A⊲\{U_{A},U_{B},T_{A},T_{B},R_{S}\}}{A|\equiv S|\sim (U_{A},U_{B},T_{A},T_{B},R_{S})} \end{array}\;\;\;\;\\ \;\;\;\;\;\;\;\; \end{array}$$

According to *M*_*SA*_, Freshness rule(*R*_4_) and *A*_*A*2_, we can obtain:

$$\begin{array}{ll} S_{18}: & \frac{A|\equiv \#(a),T_{A}=T_{a}(\alpha )}{A|\equiv \#(T_{A})},\frac{A|\equiv \#(T_{A})}{A|\equiv \#H(U_{A}∥U_{B}∥T_{A}∥T_{B}∥R_{S}∥A\overset{X_{A}}{↔}S)},\\ & \frac{A|\equiv \#H(U_{A}∥U_{B}∥T_{A}∥T_{B}∥R_{S}∥A\overset{X_{A}}{↔}S)}{A|\equiv \#(U_{A},U_{B},T_{A},T_{B},R_{S})} \end{array}\;\;\;\;$$

According to *S*_17_ and *S*_18_, we apply the Nonce-verification rule (*R*_2_) and Belief rule(*R*_5_), we can obtain:

$$\begin{array}{ll} S_{19}: & \frac{A|\equiv \#(U_{A},U_{B},T_{A},T_{B},R_{S}),A|\equiv S|\sim (U_{A},U_{B},T_{A},T_{B},R_{S})}{A|\equiv S|\equiv (U_{A},U_{B},T_{A},T_{B},R_{S})},\\ & \frac{A|\equiv S|\equiv (U_{A},U_{B},T_{A},T_{B},R_{S})}{A|\equiv S|\equiv R_{S},A|\equiv S|\equiv U_{B},A|\equiv S|\equiv T_{B}} \end{array}\;\;\;\;\;\;:Goal_{3}$$

According to *S*_19_, *A*_*A*7_ and *A*_*A*8_, we apply the Jurisdiction rule (*R*_3_), we can obtain:

$$S_{20}:\frac{A|\equiv S|\Rightarrow T_{B},A|\equiv S|\equiv T_{B}}{A|\equiv T_{B}},\frac{A|\equiv S|\Rightarrow R_{S},A|\equiv S|\equiv R_{S}}{A|\equiv R_{S}}$$

According to *S*_20_, *A*_*A*1_ and *K*_*AB*_ = *H*(*T*_*AB*_ || *R*_*S*_), we apply the Belief rule(*R*_5_), we can obtain:

$$S_{21}:\frac{A|\equiv T_{B},A|\equiv a}{A|\equiv T_{AB}(=T_{a}(T_{B}))},\frac{A|\equiv T_{AB},A|\equiv R_{S}}{A|\equiv A\overset{K_{AB}}{↔}B}:Goal_{7}$$

According to *M*_6_ and *S*_21_, we apply the Message-meaning rule (*R*_1_) and Hash function rule(*R*_8_), we can obtain:

$$\begin{array}{ll} S_{22}: & \frac{A|\equiv A\overset{K_{AB}}{↔}B,\;A⊲<H(U_{A}∥U_{B}∥T_{A}∥T_{B}∥R_{S}∥A\overset{K_{AB}}{↔}B){>}_{A\overset{K_{AB}}{↔}B}}{A|\equiv \;B|\sim H(U_{A}∥U_{B}∥T_{A}∥T_{B}∥R_{S}∥A\overset{K_{AB}}{↔}B)},\\ & \frac{A|\equiv B|\sim H(U_{A}∥U_{B}∥T_{A}∥T_{B}∥R_{S}∥A\overset{K_{AB}}{↔}B),A⊲\{U_{A},U_{B},T_{A},T_{B},R_{S}\}}{A|\equiv B|\sim A\overset{K_{AB}}{↔}B} \end{array}\;\;\;\;\;\;$$

According to *M*_6_, Freshness rule(*R*_4_) and *A*_*A*2_, we can obtain:

$$S_{23}:\frac{A|\equiv \#(a),T_{A}=T_{a}(\alpha )}{A|\equiv \#(T_{A})},\frac{A|\equiv \#(T_{A})}{A|\equiv \#A\overset{K_{AB}}{↔}B}$$

According to *S*_22_ and *S*_23_, we apply the Nonce-verification rule (*R*_2_), we can obtain:

$$S_{24}:\frac{A|\equiv \#A\overset{K_{AB}}{↔}B,A|\equiv B|\sim A\overset{K_{AB}}{↔}B}{A|\equiv B|\equiv A\overset{K_{AB}}{↔}B}:Goal_{9}$$

According to *M*_7_ and *S*_16_, we apply the Message-meaning rule (*R*_1_) and Hash function rule(*R*_8_), we can obtain:

$$\begin{array}{ll} S_{25}: & \frac{B|\equiv A\overset{K_{AB}}{↔}B,\;B⊲<H(U_{B}∥U_{A}∥T_{B}∥T_{A}∥R_{S}∥A\overset{K_{AB}}{↔}B){>}_{A\overset{K_{AB}}{↔}B}}{B|\equiv \;A|\sim H(U_{B}∥U_{A}∥T_{B}∥T_{A}∥R_{S}∥A\overset{K_{AB}}{↔}B)},\\ & \frac{B|\equiv A|\sim H(U_{B}∥U_{A}∥T_{B}∥T_{A}∥R_{S}∥A\overset{K_{AB}}{↔}B),B⊲\{U_{A},U_{B},T_{A},T_{B},R_{S}\}}{B|\equiv A|\sim A\overset{K_{AB}}{↔}B} \end{array}\;\;\;\;\;\;$$

According to *M*_7_, Freshness rule(*R*_4_) and *A*_*B*2_, we can obtain:

$$S_{26}:\frac{B|\equiv \#(b),T_{B}=T_{b}(\alpha )}{B|\equiv \#(T_{B})},\frac{B|\equiv \#(T_{B})}{B|\equiv \#A\overset{K_{AB}}{↔}B}$$

According to *S*_25_ and *S*_26_, we apply the Nonce-verification rule (*R*_2_), we can obtain:

$$S_{27}:\frac{B|\equiv \#A\overset{K_{AB}}{↔}B,B|\equiv A|\sim A\overset{K_{AB}}{↔}B}{B|\equiv A|\equiv A\overset{K_{AB}}{↔}B}:Goal_{10}$$

### 5.2 Validation test based on AVISPA

In this section, we simulate the proposed scheme for the formal security analysis using AVISPA. The AVISPA tool provides the role based HLPSL (High-Level Protocol Specification Language) for specification of protocols and security properties and four back-ends: OFMC(On-the-fly Model-Checker), CL-AtSe(Constraint-Logic-based Attack Searcher), SATMC(SAT-based ModelChecker) and TA4SP(Tree Automata-based Protocol Analyzer), which are used to identify active and inactive attacks on the protocol such as Man-In-The-Middle attack and replay attack, and to analyse various security properties of the protocol, such as key security and authentication [25, 50].

In order to verify the security properties of the protocol using AVISPA, it needs to be specified in HLPSL (High Level Protocol Specification Language).

#### Specifying the proposed protocol

**Fig 3:**
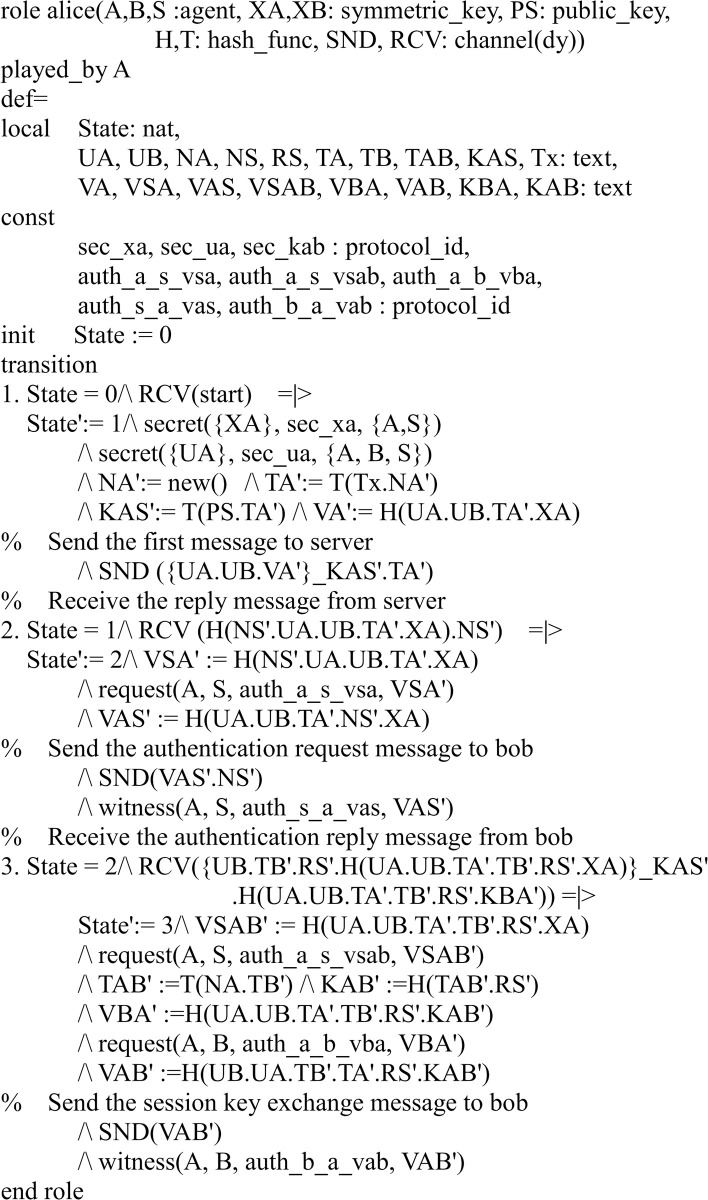
Role specification in HLPSL for the user A.

**Fig 4:**
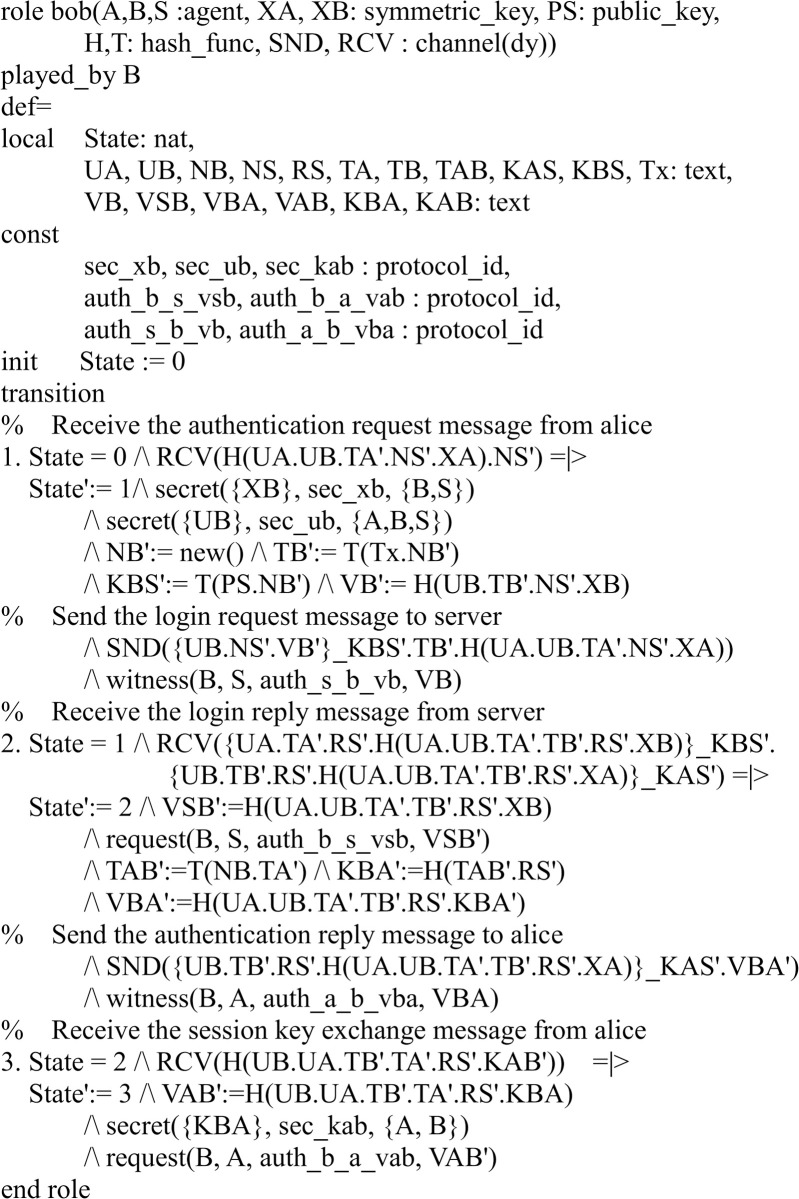
Role specification in HLPSL for the user B.

**Fig 5:**
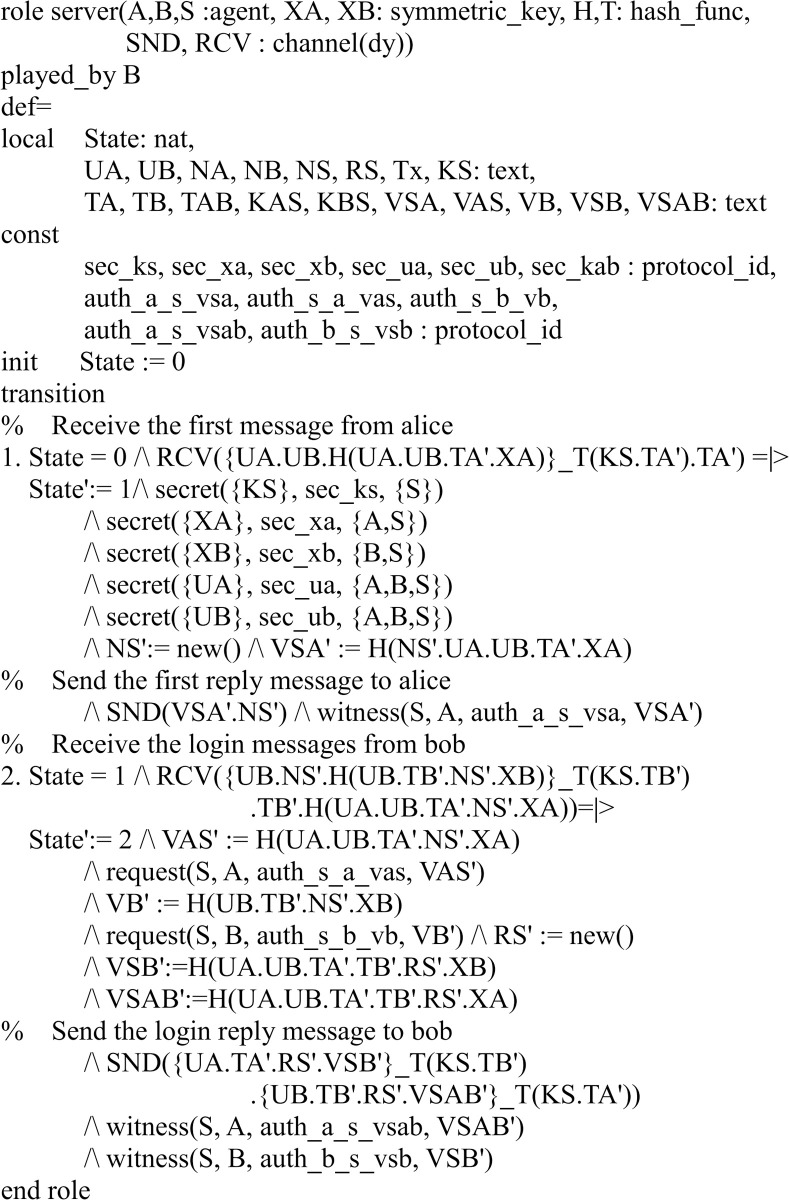
Role specification in HLPSL for the server S.

There are three participants in the proposed protocol: server *S* and two users *A*, *B*. Figs 3–5 shows the specifications in HLPSL for the role of users *A*, *B*, and server *S*.

**Fig 6:**
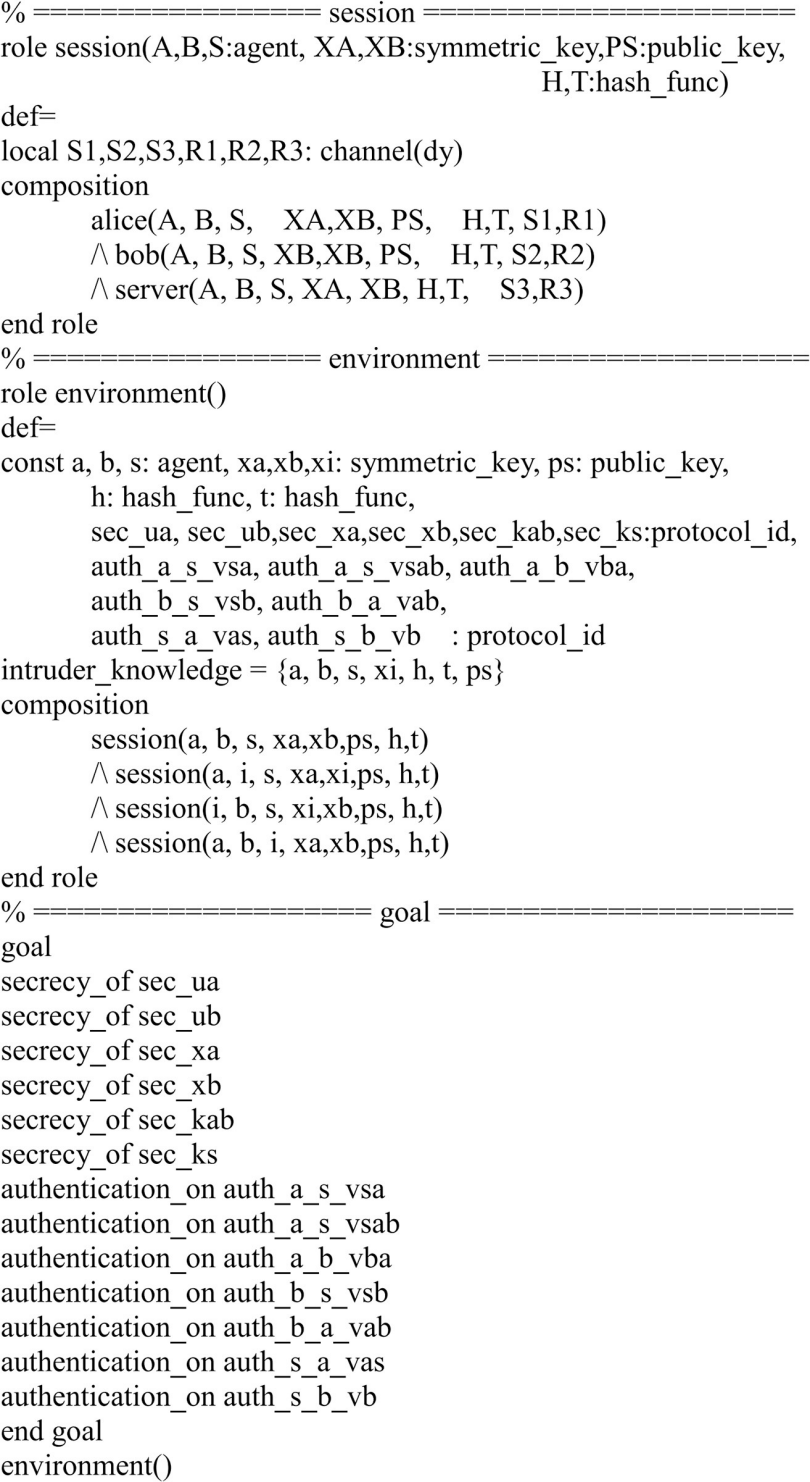
Role specification in HLPSL for the session, environment and goal.

In Fig 6, we show the HLPSL implementation for the role of the session, environment and goal.

In our implementation, we verified the six secrecy goals containing the user anonymity and the user’s secret preserving and seven authentication properties for the mutual authentication.

#### Analysis of the results

**Fig 7:**
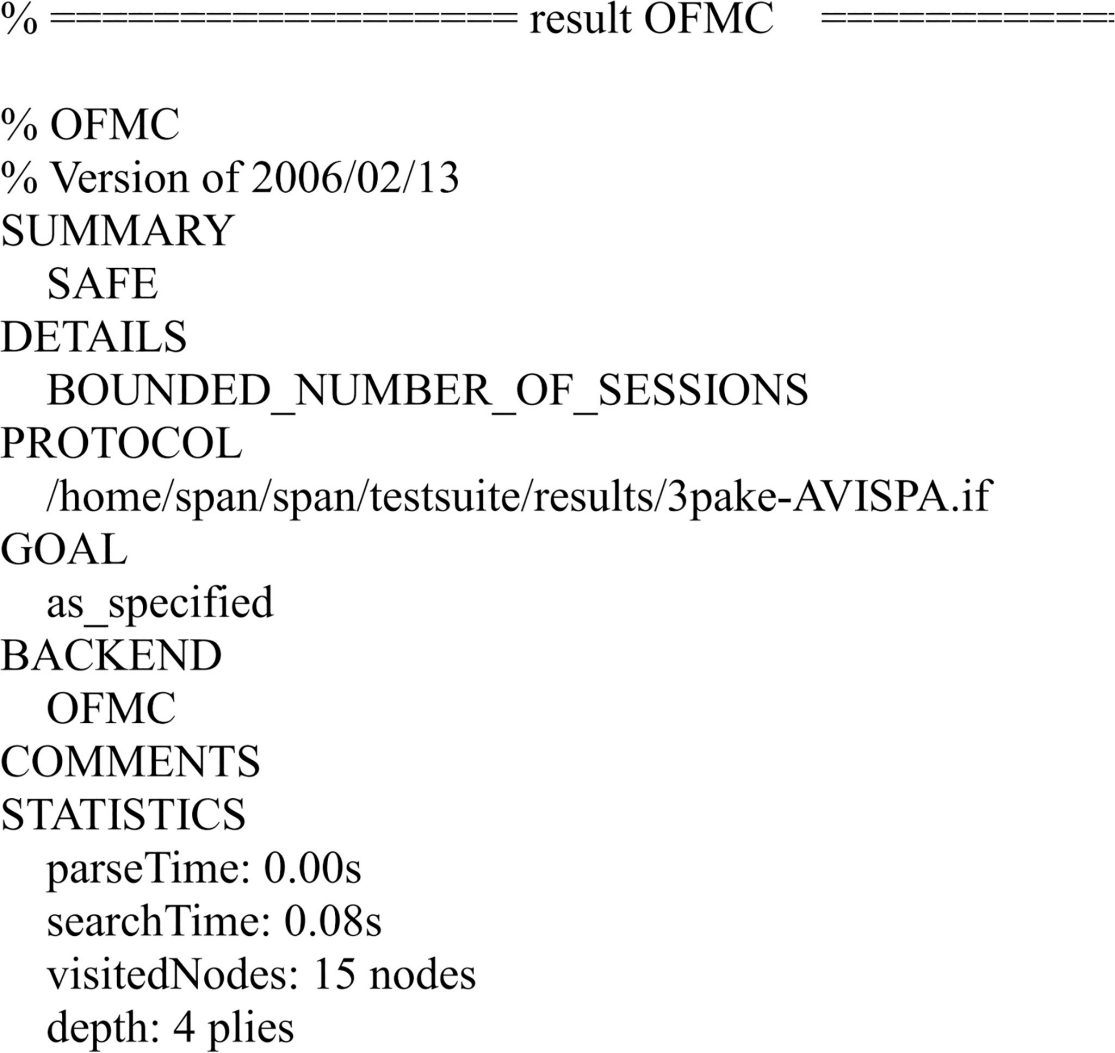
The result of the analysis using OFMC back-end.

**Fig 8:**
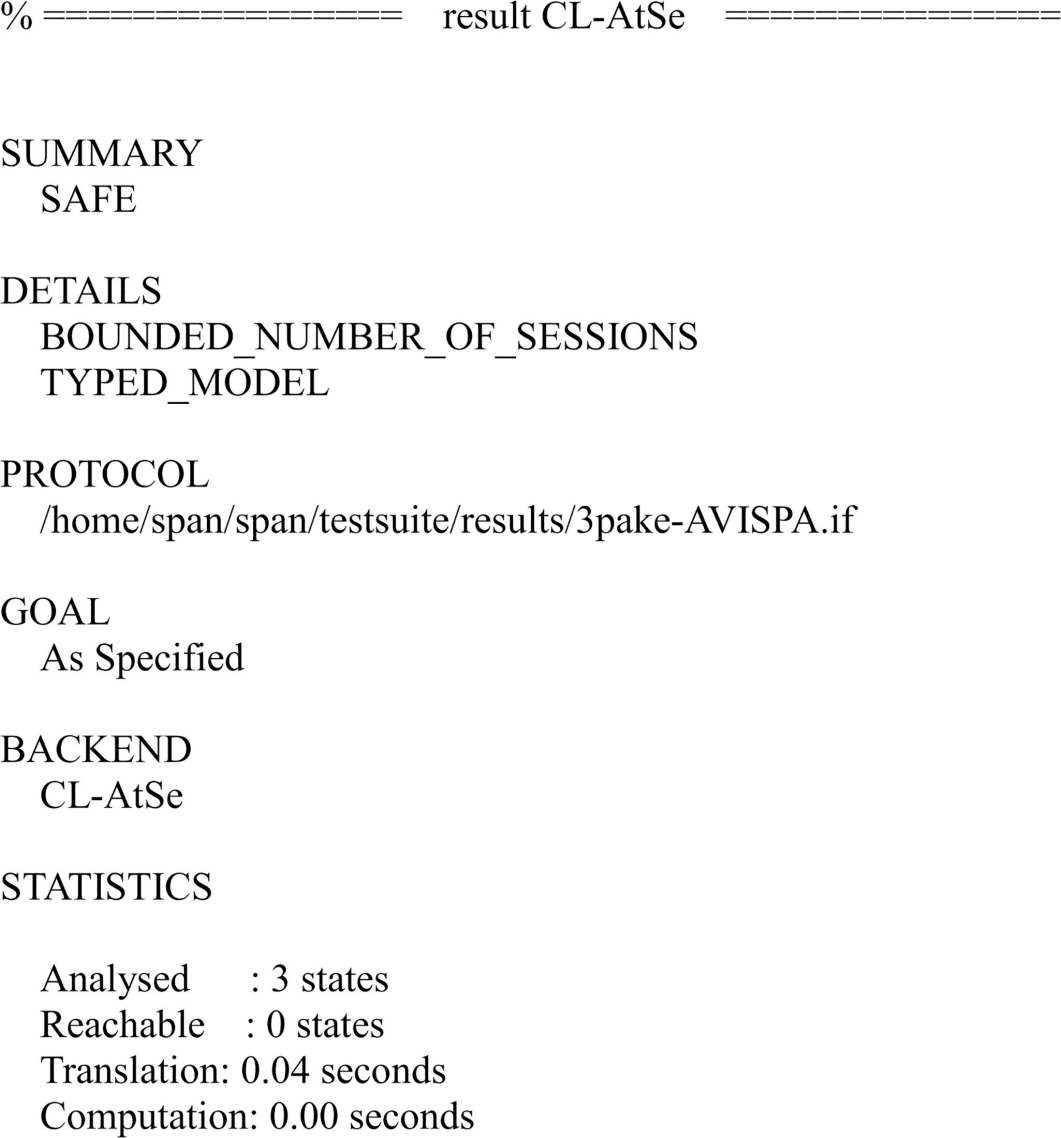
The result of the analysis using CL-AtSe back-end.

We have simulated the proposed scheme using FMC and CL-AtSe back-ends of AVISPA. The simulation results of the security verification are shown in Figs 7 and 8.

The results ensure that the proposed scheme is secure under the test of AVISPA using OFMC and CL-AtSe back-ends, and guarantees user anonymity and provides with mutual authentication, and it is also secure against the passive attacks and the active attacks, such as the replay attack and man-in-the-middle attack.

### 5.3 Informal security analysis

In this section, we demonstrate that the proposed scheme can resist various kinds of attacks and provides various security properties such as mutual authentication, user anonymity, untraceability and so on.

#### Mutual authentication

Mutual authentication is a key feature of the authenticated key agreement protocol. The proposed scheme achieves strong mutual authentication. In the proposed scheme, *X*_*i*_ is a shared secret between the server *S* and the user *U*_*i*_ in the registration phase. Also, *N*_*s*_ is a nonce of the server *S*, and *a*, *b* are secrets generated by user *A* and user *B* for generating a session key and these are also used as the nonce.

In the *Step*5 of the authentication and key exchange phase, the server *S* receives the message *M*_4_ from user *B*, which includes *V*_B_* = *H*(*U*_*B*_||*T*_*B*_||*N*_*S*_||*X*_*B*_) generated by user *B* and the *V*_AS_* = *H*(*U*_*A*_||*U*_*B*_||*T*_*A*_||*N*_*S*_||*X*_*A*_) generated by user *A*. *S* also computes *V*_*B*_ = *H*(*U*_B_*||*T*_B_*||*N*_S_*||*X*_*B*_), *V*_*AS*_ = *H*(*U*_*A*_||*U*_*B*_||*T*_*A*_||*N*_S_*||*X*_*A*_) and authenticates the user *A* and *B* through verifying whether *V*_*B*_ = *V*_B_* and *V*_*AS*_ = *V*_AS_*. Since *X*_*A*_ and *X*_*B*_ are the secrets shared between *S* and the user *A*, *B* and *N*_*S*_ is a nonce of *S*, *V*_B_* can be generated only by user *B*, and *V*_AS_* can be generated only by user *A*. Thus, *S* can authenticate the user *A* and *B* through checking these values. In the *Step*6, the user *B* receives the message *M*_5_ from *S*, which includes *V*_SB_* = *H*(*U*_*A*_||*U*_*B*_||*T*_*A*_||*T*_*B*_||*R*_*S*_||*X*_*B*_) generated by the server *S*. Since *T*_*B*_ (= *T*_*b*_(*α*) *mod p*) includes *B*’s nonce *b* and *X*_*B*_ is a secret shared with *S*, *V*_SB_* can be generated only by *S*. Therefore, *B* can authenticate *S* through checking *V*_SB_*. In the *Step*7, the user *A* also can authenticate *S* through verifying *V*_SAB_* = *H*(*U*_*A*_||*U*_B_*||*T*_*A*_ (= *T*_*a*_(*α*) *mod p*)||*T*_B_*||*R*_S_*|*X*_*A*_). Likewise, User *A* authenticates User *B* through checking *V*_*BA*_ = *H*(*U*_*A*_||*U*_B_*||*T*_*A*_||*T*_B_*||*R*_S_*||*K*_*AB*_) at *Stage*7, and User *B* authenticates User *A* through checking *V*_*AB*_ = *H*(*U*_*B*_|| *U*_*A*_|| *T*_*B*_|| *T*_*A*_||*R*_*S*_||*K*_*AB*_) at *Stage*8.

#### User anonymity

The proposed scheme guarantees user anonymity. The messages (*M*_*AS*_, *M*_*BS*_, *M*_*SA*_ and *M*_*SB*_) associated with the user’s identifier are encrypted with the secret key, which is only known for each participant. For example, *M*_*AS*_ is encrypted with the secret key *K*_*AS*_, which is calculated as follows: *K*_*AS*_ = *T*_*a*_(*T*_*s*_(*x*)) = *T*_*s*_(*T*_*a*_(*x*)), where the random number *a* is only known for the user *A* and the secret key *s* is only known for the server *S*.

Even if *T*_*a*_(*x*) and *T*_*s*_(*x*) is exposed, according to *CDLP* and *CDHP* assumptions, it is impossible for a third party to calculate *K*_*AS*_ or *a*, *s*. Therefore, a third party except user and server cannot know the user’s identifier.

#### Untraceability

The proposed protocol provides untraceability.

As in the user anonymity proof, all messages (*M*_*AS*_, *M*_*BS*_, *M*_*SA*_ and *M*_*SB*_) containing the user’s identity are encrypted as follows.

*M*_*AS*_ = *E*_*KAS*_(*U*_*A*_, *U*_*B*_, *V*_*A*_), *M*_*SA*_ = *E*_*KAS*_(*U*_*B*_, *T*_*B*_, *R*_*S*_, *V*_*SAB*_), *K*_*AS*_ = *T*_*ks*_(*T*_*A*_) = *T*_*ksa*_(*α*) *mod p*
*M*_*BS*_ = *E*_*KBS*_(*U*_*B*_, *N*_*S*_, *V*_*B*_), *M*_*SB*_ = *E*_*KBS*_(*U*_*A*_, *T*_*A*_, *R*_*S*_, *V*_*SB*_), *K*_*BS*_ = *T*_*ks*_(*T*_*B*_) = *T*_*bks*_(*α*) *mod p*

Then, the encryption keys *K*_*AS*_ and *K*_*BS*_ are all computed from the random numbers *a* and *b* generated by user *A* and *B*, so that different messages are exchanged in different sessions. Other messages also contain a random number in different sessions, so that they are presented random bit arrays in each sessions.

Therefore, the proposed protocol provides untraceability.

### Off-line password guessing attack

The proposed scheme is secure against the password guessing attack.

In the proposed scheme, user’s password is used for accessing the smart card and the information related to it does not disclose in public channel.

The information stored in the user *A*’s smart card is {*p*, *α*, *P*_*s*_, *G*_*A*_, *F*_*A*_, *T*_*n*_(∙), *H*(∙), *E*_*K*_(∙), *D*_*K*_(∙)}, and the information related to the user’s password is *G*_*A*_ = *H*(*U*_*A*_||*pw*_*A*_||*h*(*bm*_*A*_))⊕*X*_*A*_ and *F*_*A*_ = *H*(*U*_*A*_||*pw*_*A*_||*h*(*bm*_*A*_) ||*X*_*A*_). Suppose that an attacker steals user *A*’s smart card *SC*_*A*_ and knows his identifier *U*_*A*_. In order to guess the user *A*’s password, the attacker must compute *PR*_A_* = *H*(*U*_*A*_||*pw*_A_*||*h*(*bm*_*A*_)), *X*_A_* = *G*_*A*_ ⊕ *PR*_A_* and *F*_A_* = *H*(*U*_*A*_||*pw*_A_*||*h*(*bm*_*A*_)||*X*_A_*) with any password *pw*_A_* to compare *F*_A_* and *F*_*A*_ stored in *SC*_*A*_. However, as the attacker cannot know *h*(*bm*_*A*_), he cannot compute *PR*_A_*. Therefore, the attacker cannot guess the user’s password.

#### Privileged insider attack

The proposed scheme is secure against the privileged-insider attack. In the proposed scheme, user’s password is not transmitted to the server *S* and the privilege insider of the server cannot know the user’s password. Therefore, the proposed scheme is secure against this attack.

#### Stolen verifier attack

The proposed scheme is secure against stolen verifier attack. In the registration phase of the proposed scheme, the server stores a tuple {*U*_*A*_, *N*_*a*_} into his user register-table, where *U*_*A*_ is user *A*’s identifier and *N*_*a*_ is a random number selected by the server. These are not sensitive to authenticate the user. Therefore, the proposed scheme is secure against stolen verifier attack.

#### User impersonate attack

The proposed scheme is secure against the user impersonate attack.

The user impersonate attack is only possible in the scheme which can’t provide a certain authentication. For example, if a participant *X* can’t authenticate a participant *Y*, an attacker can impersonate as *Y*. As shows the above, the proposed scheme achieves certain mutual authentication. In the *Step*5, the server *S* certainly authenticates *A* and *B* with his nonce *N*_*S*_ and the user’s secret *X*_*A*_ and *X*_*B*_. If an attacker wants to impersonate as the user *A*, he must compute *V*_*A*_ = *H*(*U*_*A*_||*U*_*B*_||*T*_*A*_||*X*_*A*_) or *V*_*AS*_ = *H*(*U*_*A*_||*U*_*B*_||*T*_*A*_||*N*_*S*_||*X*_*A*_), but he doesn’t know the user *A*’s secret *X*_*A*_ = *H*(*U*_*A*_||*N*_*a*_||*k*_*s*_) and could not compute it (Because *k*_*s*_ is the server’s secret key), so he cannot compute *V*_*A*_ or *V*_*AS*_ and cannot impersonate as the user *A*. As the same, an attacker cannot impersonate as the user *B*.

#### Man-in-the-middle attack

As shows the above, the proposed scheme achieves certain mutual authentication, so an attacker cannot impersonate as the initiator *A* and the responder *B*, and cannot achieve the man-in-the-middle attack.

If an attacker wants to achieve a man-in-the-middle attack, he must exchange a session key *K*_AB_* = *H*(*T*_*AB*_ ||*R*_S_*) with users *A* and *B*.

Suppose that an attacker generates a random number b* and *R*_S_* to exchange the session key with user *A*, computes *T*_AB_* = *T*_b*_(*T*_*a*_) = *T*_ab*_(*α*) *mod p*, and then computes *K*_AB_* = *H*(*T*_AB*_ ||*R*_S_*). However, the attacker cannot generate *V*_*SAB*_ = *H*(*U*_*A*_||*U*_B_*||*T*_*A*_||*T*_B_*||*R*_S_*||*X*_*A*_) because he does not know the user *A*’s secret *X*_*A*_. Therefore, in *Step* 7, user *A* can detect the attack via checking for *V*_*SAB*_.

To exchange the session key with user *B*, an attacker has to compute *K*_AB_* by generating random numbers a* and *R*_S_* and calculate *V*_*SB*_ = *H*(*U*_A_*||*U*_*B*_||*T*_A_*||*T*_*B*_||*R*_S_*||*X*_*B*_). However, since the attacker does not know the user *B*’s secret *X*_*B*_, it is impossible to compute the *V*_*SB*_, so in *Step* 6, user *B* can detect the attack via checking for the *V*_*SB*_.

That is, the proposed scheme is resistant to man-in-the-middle attack.

#### Replay attack

In the *Step*5 of the proposed scheme, the server *S* certainly authenticates *A* and *B*.

If an attacker *C* intercepts the previous message *M*_1_ = {*M*_AS_*, *T*_A_*} of the user *A* and retransmits it to the server, the server responses *M*_2_ = {*V*_*SA*_, *N*_*S*_}, where *N*_*S*_ is generated by the server. However, in the *Step*3, the attacker must compute *M*_3_ = {*V*_*AS*_, *N*_*S*_}, but he does not know the user *A*’s secret *X*_*A*_ and he cannot compute *V*_*AS*_ = *H*(*U*_*A*_||*U*_*B*_||*T*_*A*_||*N*_*S*_||*X*_*A*_). Therefore, in the *Step*5, the server *S* can successfully detect the replay attack through checking for *V*_*AS*_. Likewise, if an attacker retransmits the user *B*’s message *M*_4_ to server *S*, in the *Step*5, the server can detect the replay attack through checking for *V*_*B*_ = *H*(*U*_B_*||*T*_B_*||*N*_S_*||*X*_*B*_) containing the user *B*’s secret *X*_*B*_.

#### Known key security

In the proposed scheme, the session key *K*_*AB*_ is calculated as *K*_*AB*_ = *H*(*T*_*ab*_(*α*) *mod p* || *R*_*S*_). It contains the random numbers *a*, *b* and *R*_*S*_ that are generated by each participant for each session. Even if an attacker knows the previous session key, he cannot calculate a new session key.

## 6. Performance comparisons

This section, we evaluate the computational cost, communication overhead and security performance of our proposed scheme and other recent 3PAKE schemes [19, 22, 23, 61]. For comparison of computational cost, we define some notations as follows.

t_c_: time needed for Chebyshev polynomial operation
t_h_: time needed for one-way hash function operation
t_m_: time needed for a modular exponential operation
t_r_: time needed for a modular squaring operation
t_q_: time needed for a square root modulo N operation
t_s_: time needed for symmetric encryption/decryption operation

Table 4 shows the comparison of the computational cost of our proposed scheme and other 3PAKE schemes. As shown Table 4, the computational cost of our proposed scheme is lower than Jabbari et al.’s scheme, Li et al.’s scheme and Lu et al.’s scheme.

**Table 4:** Comparison of the computational cost between the proposed scheme and other 3PAKE schemes.

	Irshad et al. [61]	Jabbari et al. [19]	Li et al. [22]	Lu et al. [23]	proposed
A	3t_c_ + 10t_h_	4t_c_ + 2t_s_ + 4t_h_	4t_c_ + 1t_r_ + 5t_h_	3t_c_ + 4t_s_ + 4t_h_	3t_c_ + 2t_s_ + 9t_h_
B	3t_c_ + 1t_s_ + 10t_h_	3t_c_ + 2t_s_ + 4t_h_	4t_c_ + 1t_r_ + 5t_h_	2t_c_ + 3t_s_ + 5t_h_	3t_c_ + 2t_s_ + 7t_h_
S	2t_c_ + 1t_s_ + 10t_h_	4t_c_ +4t_s_ + 4t_h_	4t_c_ + 2t_q_ + 5t_h_	5t_c_ + 5t_s_+ 7t_h_	2t_c_ + 4t_s_ + 8t_h_
Total	8t_c_ + 2t_s_ + 30t_h_	11t_c_ + 8t_s_ + 12t_h_	12t_c_+2t_m_+2t_q_+15t_h_	10t_c_ + 12t_s_+ 16t_h_	8t_c_ + 8t_s_ + 24t_h_
Round	5	5	6	6	7

In order to presume the communication overhead of our proposed scheme, we consider the bit size of identity, random number |N|, timestamp, hash(SHA-1) output and Chebyshev chaotic maps as |ID| = 160, |N| = 160, |Ts| = 32, |H| = 160, |CM| = 160 bits, respectively. Furthermore, the bit sizes used for modular exponentiation and modular square operations are considered as |ME| = 1024 bits [25]. Table 5 shows the communication overhead of our proposed scheme according to above assumption.

**Table 5:** Communication overhead of our proposed scheme.

	Expression	Length of message
M1	2|ID| + |H| + |T|	640
M2	|H| +|N|	320
M3	|H| +|N|	320
M4	|ID| + |N| + 2|H| + |T|	800
M5	2|ID| + 2|N| + 2|H| + 2|T|	1280
M6	|ID| + |N| + 2|H| + |T|	800
M7	|H|	160
Total	6|ID| + 6|N| + 10|H| + 5|T|	4320

Table 6 shows the comparison of the communication overhead of our proposed scheme and other 3PAKE schemes. As shown Table 6, our proposed scheme has many message rounds and its communication overhead is higher than other schemes.

**Table 6:** Comparison of the computational cost between the proposed scheme and other 3PAKE schemes.

	Irshad et al. [61]	Jabbari et al. [19]	Li et al. [22]	Lu et al. [23]	proposed
Expression	|Ts|+14|H| +6|T|	11|ID|+8|H| +9|T|	2|ME|+6|H|+5|T|	6|ID|+7|H|+7|T|	6|ID|+6|N|+ 10|H| + 5|T|
Bits	3232	4480	3808	3200	4320

Table 7 shows the comparative evaluation of the security function between the proposed scheme and other 3PAKE schemes.

**Table 7:** Comparative evaluation of the security function between the proposed scheme and other 3PAKE schemes.

	Irshad et al. [61]	Jabbari et al. [19]	Li et al. [22]	Lu et al. [23]	proposed
Mutual authentication	Yes	Yes	Yes	Yes	Yes
Provision of User anonymity	Yes	Yes	No	No	Yes
Provision of untraceability	No	Yes	No	No	Yes
Protection of Privileged insider attack	Yes	Yes	Yes	Yes	Yes
Protection of stolen verifier attack	Yes	Yes	No	Yes	Yes
Protection of User impersonate attack	Yes	Yes	No	Yes	Yes
Protection of verifier disclose attack	Yes	Yes	No	Yes	Yes
Provision of Password change phase	Yes	Yes	Yes	Yes	Yes
Provision of re-registration phase	No	No	No	No	Yes
Without timestamp	Yes	Yes	Yes	Yes	Yes
Using smart card	Yes	No	No	No	Yes

As shown in Table 7, the proposed scheme outperforms the other schemes in terms of the security functions presented.

Irshad’s scheme has lower communication overhead and computational cost than the proposed scheme, but it does not provide untraceability and re-registration phase.

Jabbari’s scheme has higher communication overhead and more expensive computational costs than ours and it does not provide re-registration phase.

Li’s scheme has lower communication overhead than ours, but it has more expensive computational costs and his scheme attempted to provide user anonymity, but did not achieve it. His scheme is also vulnerable to the verifier disclose attack, user impersonate attack and stolen verifier attack and it has faults in password change phase.

Lu’s scheme has lower communication overhead than ours, but it has more expensive computational costs and his scheme does not provide user anonymity, untraceability and re-registration phase.

## 7. Conclusion and future work

In this paper, we have analysed the Li et al.’s scheme and proved that his scheme has some faults, and proposed an enhanced three-party mutual authentication key exchange(3PMAKE) protocol based on chaotic maps using smart card to provide with user anonymity and untraceability in the environment for user-to-user communication. The proposed scheme provides strong mutual authentication between servers and users without using timestamp, can be re-registered to the system without changing the user’s identifier. The proposed scheme also provides anonymity and untraceability and is secure against several attacks such as user impersonate attacks, privileged insider attacks, stolen verifier attacks. In addition, we have formally analysed the security properties of proposed scheme and verified their validity based on BAN logic and AVISPA tool, and proved that the proposed scheme is secure against various attacks through informal security analysis.

The proposed scheme is designed to provide strong mutual authentication between communication participants without a timestamp, so the number of message exchanges and communication overhead are relatively high. In addition, since key exchange is performed based on chaotic-maps, the security performance of the proposed scheme is enhanced, but has the limitation of increasing computational cost compared to lightweight schemes that do not use public key encryption. The proposed method is suitable for systems that have to provide stronger security properties in environments where timestamp is not available and there is no restriction on communication overhead.

In the future, we will investigate more improved authentication key exchanges in IoT or WSN environments that requires lightweight scheme in terms of communication overhead or computational cost. That is, instead of public key encryption, we only use hash functions to reduce the computational cost of key exchange and reduce the communication overhead.

## Supporting information

S1 Protocol (DOCX)
