# Supplementary material for: Improved anonymity preserving three-party mutual authentication key exchange protocol based on chaotic maps
Source: PLoS One. 2022 Sep 16;17(9):e0273664. doi: 10.1371/journal.pone.0273664 (PMC9481006; doi:10.1371/journal.pone.0273664)
Supplement: S1 Protocol — (DOCX) [file pone.0273664.s001.docx]

| User *A*/*SC_A_* Server *S* |
| --- |
| {*U_A_* }  *X_A_* = *H*(*U_A_*\|\|*N_a_*\|\|*k_s_*)  *SC_A_*←{*p*,*α*,*P_s_*,*X_A_*,*T_n_*(∙),*H*(∙),*E_K_*(∙),*D_K_*(∙)}  *SC_A_*  Input *pw_A ,_ bm_A_*  *G_A_* = *H*(*U_A_*\|\|*pw_A_*\|\|*h*(*bm_A_*)) ⊕ *X_A_*  *F_A_* = *H*(*U_A_*\|\|*pw_A_*\|\| *h*(*bm_A_*)\|\|*X_A_*)  *SC_A_*← {*p*, *α*, *P_s_*, *G_A_*, *F_A_*, *T_n_*(∙), *H*(∙), *E_K_*(∙), *D_K_*(∙)} |

| User *A*/*SC_A_* Server *S* User *B*/*SC_B_* | User *A*/*SC_A_* Server *S* User *B*/*SC_B_* |
| --- | --- |

| 1) Inserts *SC_A_*, inputs *U_A_*, *pw_A_*, *bm_A_*  *X_A_*^*^ = *G_A_* ⊕ *H*(*U_A_*\|\|*pw_A_*\|\|*h*(*bm_A_*))  *F_A_*^*^ = *H*(*U_A_*\|\|*pw_A_*\|\|*h*(*bm_A_*)\|\|*X_A_*^*^).  If (*F_A_* ≠ *F_A_*^*^), aborts the process  *T_A_* = *T_a_*(*α*) *mod p*, *K_AS_* = *T_a_*(*P_S_*) = *T_aks_*(*α*) *mod p*,  *V_A_* = *H*(*U_A_*\|\|*U_B_*\|\|*T_A_*\|\|*X_A_*), *M_AS_* = *E_KAS_*(*U_A_*, *U_B_*, *V_A_*).  *M*_1_ = {*M_AS_*, *T_A_*} |  |
| --- | --- |

|  | 2) *K_AS_* = *T_ks_*(*T_A_*), {*U_A_*^*^, *U_B_*^*^, *V_A_*^*^} = *D_KAS_* (*M_AS_*)  *X_A_* = *H*(*U_A_^*^*\|\|*N_a_*\|\|*k_s_*), *V_A_* = *H*(*U_A_*^*^\|\|*U_B_*^*^\|\|*T_A_*^*^\|\|*X_A_*).  If *V_A_* ≠ *V_A_*^*^, *S* aborts the process.  *V_SA_* = *H*(*N_S_*\|\|*U_A_*\|\|*U_B_*\|\| *T_A_*\|\|*X_A_*)  *M*_2_ = { *V_SA_*, *N_S_* } |
| --- | --- |

| 3) *V_SA_* = *H*(*N_S_*^*^\|\|*U_A_*\|\|*U_B_*\|\| *T_A_*\|\|*X_A_*).  If *V_SA_* ≠ *V_SA_*^*^, *A* aborts the process.  *V_AS_* = *H*(*U_A_*\|\|*U_B_*\|\|*T_A_*\|\|*N_S_*\|\|*X_A_*),  *M*_3_ = {*V_AS_*, *N_S_* } |  |
| --- | --- |

|  | 4) Inserts*SC_B_*, inputs *U_B_*, *pw_B_*, *bm_B_*  *X_B_*^*^ = *G_B_* ⊕ *H*(*U_B_*\|\|*pw_B_*\|\|*h*(*bm_B_*)),  *F_B_*^*^ = *H*(*U_B_*\|\|*pw_B_*\|\|*h*(*bm_B_*)\|\|*X_B_*^*^).  If *F_B_* ≠ *F_B_^*^*, *SC_B_* aborts the process  *T_B_* = *T_b_*(*α*) *mod p*, *K_BS_* = *T_b_*(*P_S_*) = *T_bks_*(*α*) *mod p*,  *V_B_* = *H*(*U_B_*\|\|*T_B_*\|\|*N_S_*\|\|*X_B_*), *M_BS_* = *E_KBS_*(*U_B_*, *N_S_*, *V_B_*).  *M*_4_= {*M_BS_*, *T_B_*, *V_AS_* } |
| --- | --- |

|  | 5) *K_BS_* = *T_ks_*(*T_B_*), {*U_B_*^*^, *N_S_*^*^, *V_B_*^*^} = *D_KBS_* (*M_BS_*)  *X_B_* = *H*(*U_B_^*^*\|\|*N_b_*\|\|*k_s_*), *V_B_* = *H*(*U_B_*^*^\|\|*T_B_*^*^\|\|*N_S_*^*^\|\|*X_B_*).  *V_AS_* = *H*(*U_A_*\|\|*U_B_*\|\|*T_A_*\|\|*N_S_*^*^\|\|*X_A_*)  If *V_B_* ≠ *V_B_*^*^ or *V_AS_* ≠ *V_AS_* ^*^, *S* aborts the process.  *S* selects a random number *R_S_*  *V_SB_* = *H*(*U_A_*\|\|*U_B_*\|\|*T_A_*\|\|*T_B_*\|\|*R_S_*\|\|*X_B_*), *M_SB_* = *E_KBS_*(*U_A_*, *T_A_*, *R_S_*, *V_SB_*)  *V_SAB_* = *H*(*U_A_*\|\|*U_B_*\|\|*T_A_*\|\|*T_B_*\|\|*R_S_*\|\|*X_A_*), *M_SA_* = *E_KAS_*(*U_B_*, *T_B_*, *R_S_*, *V_SAB_* )  *M*_5_ = {*M_SB_*, *M_SA_* } |
| --- | --- |

| *M*_6_ = {*V_BA_*, *M_SA_* } | 6) {*U_A_*^*^, *T_A_*^*^, *R_S_*^*^, *V_SB_*^*^)} = *D_KBS_* (*M_SB_*)  *V_SB_* = *H*(*U_A_*^*^\|\|*U_B_*\|\|*T_A_*^*^\|\|*T_B_*\|\|*R_S_*^*^\|\|*X_B_*)  If *V_SB_* ≠ *V_SB_* ^*^, *B* aborts the process.  *T_BA_* = *T_b_*(*T_A_*^*^)= *T_ab_*(*α*) *mod p*, *K_BA_* = *H*(*T_BA_* \|\|*R_S_*^*^)  *V_BA_* = *H*(*U_A_*^*^\|\|*U_B_*\|\|*T_A_*^*^\|\|*T_B_*\|\|*R_S_*^*^\|\|*K_BA_*) |
| --- | --- |

| 7) {*U_B_*^*^, *T_B_*^*^, *R_S_*^*^, *V_SAB_*^*^} = *D_KAS_*(*M_SA_*)  *V_SAB_* = *H*(*U_A_*\|\|*U_B_*^*^\|\|*T_A_*\|\|*T_B_*^*^\|\|*R_S_*^*^\|\|*X_A_*)  *T_AB_* = *T_a_*(*T_B_*^*^) = *T_ab_*(*α*) *mod p*, *K_AB_* = *H*(*T_AB_* \|\|*R_S_*^*^)  *V_BA_* = *H*(*U_A_*\|\|*U_B_*^*^\|\|*T_A_*\|\|*T_B_*^*^\|\|*R_S_*^*^\|\|*K_AB_*)  If *V_SAB_* ≠ *V_SAB_^*^* or *V_BA_* ≠ *V_BA_*^*^, *A* aborts the process.  *V_AB_* = *H*(*U_B_*^*^\|\|*U_A_*\|\| *T_B_*^*^\|\| *T_A_*\|\| *R_S_*^*^\|\|*K_AB_*)  *M*_7_ = {*V_AB_* } |  |
| --- | --- |

*K_AB_* is a session key

|  | 8) *V_AB_* = *H*(*U_B_*\|\|*U_A_*\|\| *T_B_*\|\| *T_A_*\|\| *R_S_*\|\|*K_BA_*)  If *V_AB_*≠*V_AB_* ^*^, *B* aborts the process.  *K_AB_* is a session key |
| --- | --- |

% ======================= alice ===========================

role alice(A,B,S :agent, XA,XB: symmetric_key, PS: public_key, H,T: hash_func,

SND, RCV: channel(dy))

played_by A

def=

local State: nat,

UA, UB, NA, NS, RS, Tx: text,

TA, TB, TAB, KAS, VA, VSA, VAS, VSAB, VBA, VAB, KBA, KAB: text

const

sec_xa, sec_ua, sec_kab : protocol_id,

auth_a_s_vsa, auth_a_s_vsab, auth_a_b_vba,

auth_s_a_vas, auth_b_a_vab : protocol_id

init State := 0

transition

1. State = 0/\ RCV(start) =|>

State':= 1/\ secret({XA}, sec_xa, {A,S})

/\ secret({UA}, sec_ua, {A, B, S})

/\ NA':= new()

/\ TA':= T(Tx.NA')

/\ KAS':= T(PS.TA')

/\ VA':= H(UA.UB.TA'.XA)

% Send the first message to server

/\ SND ({UA.UB.VA'}_KAS'.TA')

% Receive the reply message from server

2. State = 1/\ RCV (H(NS'.UA.UB.TA'.XA).NS') =|>

State':= 2/\ VSA' := H(NS'.UA.UB.TA'.XA)

/\ request(A, S, auth_a_s_vsa, VSA')

/\ VAS' := H(UA.UB.TA'.NS'.XA)

% Send the authentication request message to bob

/\ SND(VAS'.NS')

/\ witness(A, S, auth_s_a_vas, VAS')

% Receive the authentication reply message from bob

3. State = 2/\ RCV({UB.TB'.RS'.H(UA.UB.TA'.TB'.RS'.XA)}_KAS'

.H(UA.UB.TA'.TB'.RS'.KBA')) =|>

State':= 3/\ VSAB' := H(UA.UB.TA'.TB'.RS'.XA)

/\ request(A, S, auth_a_s_vsab, VSAB')

/\ TAB' :=T(NA.TB')

/\ KAB' :=H(TAB'.RS')

/\ VBA' :=H(UA.UB.TA'.TB'.RS'.KAB')

/\ request(A, B, auth_a_b_vba, VBA')

/\ VAB' :=H(UB.UA.TB'.TA'.RS'.KAB')

% Send the session key exchange message to bob

/\ SND(VAB')

/\ witness(A, B, auth_b_a_vab, VAB')

end role

% ======================= bob ===========================

role bob(A,B,S :agent, XA, XB: symmetric_key, PS: public_key, H,T: hash_func,

SND, RCV : channel(dy))

played_by B

def=

local State: nat,

UA, UB, NB, NS, RS, Tx: text,

TA, TB, TAB, KAS, KBS, VB, VSB, VBA, VAB, KBA, KAB: text

const

sec_xb, sec_ub, sec_kab : protocol_id,

auth_b_s_vsb, auth_b_a_vab, auth_s_b_vb, auth_a_b_vba : protocol_id

init State := 0

transition

% Receive the authentication request message from alice

1. State = 0 /\ RCV(H(UA.UB.TA'.NS'.XA).NS') =|>

State':= 1/\ secret({XB}, sec_xb, {B,S})

/\ secret({UB}, sec_ub, {A,B,S})

/\ NB':= new()

/\ TB':= T(Tx.NB')

/\ KBS':= T(PS.NB')

/\ VB':= H(UB.TB'.NS'.XB)

% Send the login request message to server

/\ SND({UB.NS'.VB'}_KBS'.TB'.H(UA.UB.TA'.NS'.XA))

/\ witness(B, S, auth_s_b_vb, VB)

% Receive the login reply message from server

2. State = 1 /\ RCV({UA.TA'.RS'.H(UA.UB.TA'.TB'.RS'.XB)}_KBS'.

{UB.TB'.RS'.H(UA.UB.TA'.TB'.RS'.XA)}_KAS') =|>

State':= 2 /\ VSB':=H(UA.UB.TA'.TB'.RS'.XB)

/\ request(B, S, auth_b_s_vsb, VSB')

/\ TAB':=T(NB.TA')

/\ KBA':=H(TAB'.RS')

/\ VBA':=H(UA.UB.TA'.TB'.RS'.KBA')

% Send the authentication reply message to alice

/\ SND({UB.TB'.RS'.H(UA.UB.TA'.TB'.RS'.XA)}_KAS'.VBA')

/\ witness(B, A, auth_a_b_vba, VBA)

% Receive the session key exchange message from alice

3. State = 2 /\ RCV(H(UB.UA.TB'.TA'.RS'.KAB')) =|>

State':= 3 /\ VAB':=H(UB.UA.TB'.TA'.RS'.KBA)

/\ secret({KBA}, sec_kab, {A, B})

/\ request(B, A, auth_b_a_vab, VAB')

end role

% ======================= server ===========================

role server(A,B,S :agent, XA, XB: symmetric_key, H,T: hash_func,

SND, RCV : channel(dy))

played_by B

def=

local State: nat,

UA, UB, NA, NB, NS, RS, Tx, KS: text,

TA, TB, TAB, KAS, KBS, VSA, VAS, VB, VSB, VSAB: text

const

sec_ks, sec_xa, sec_xb, sec_ua, sec_ub, sec_kab : protocol_id,

auth_a_s_vsa, auth_s_a_vas, auth_s_b_vb,

auth_a_s_vsab, auth_b_s_vsb : protocol_id

init State := 0

transition

% Receive the first message from alice

1. State = 0 /\ RCV({UA.UB.H(UA.UB.TA'.XA)}_T(KS.TA').TA') =|>

State':= 1/\ secret({KS}, sec_ks, {S})

/\ secret({XA}, sec_xa, {A,S})

/\ secret({XB}, sec_xb, {B,S})

/\ secret({UA}, sec_ua, {A,B,S})

/\ secret({UB}, sec_ub, {A,B,S})

/\ NS':= new()

/\ VSA' := H(NS'.UA.UB.TA'.XA)

% Send the first reply message to alice

/\ SND(VSA'.NS')

/\ witness(S, A, auth_a_s_vsa, VSA')

% Receive the login messages from bob

2. State = 1 /\ RCV({UB.NS'.H(UB.TB'.NS'.XB)}_T(KS.TB')

.TB'.H(UA.UB.TA'.NS'.XA)) =|>

State':= 2 /\ VAS' := H(UA.UB.TA'.NS'.XA)

/\ request(S, A, auth_s_a_vas, VAS')

/\ VB' := H(UB.TB'.NS'.XB)

/\ request(S, B, auth_s_b_vb, VB')

/\ RS' := new()

/\ VSB':=H(UA.UB.TA'.TB'.RS'.XB)

/\ VSAB':=H(UA.UB.TA'.TB'.RS'.XA)

% Send the login reply message to bob

/\ SND({UA.TA'.RS'.VSB'}_T(KS.TB').{UB.TB'.RS'.VSAB'}_T(KS.TA'))

/\ witness(S, A, auth_a_s_vsab, VSAB')

/\ witness(S, B, auth_b_s_vsb, VSB')

end role

% ======================= session ===========================

role session(A,B,S:agent, XA,XB:symmetric_key,PS:public_key, H,T:hash_func)

def=

local S1,S2,S3,R1,R2,R3: channel(dy)

composition

alice(A, B, S, XA,XB, PS, H,T, S1,R1)

/\ bob(A, B, S, XB,XB, PS, H,T, S2,R2)

/\ server(A, B, S, XA, XB, H,T, S3,R3)

end role

% ======================= environment ===========================

role environment()

def=

const a, b, s: agent, xa,xb,xi: symmetric_key, ps: public_key,

h: hash_func,

t: hash_func,

sec_ua, sec_ub, sec_xa, sec_xb, sec_kab, sec_ks : protocol_id,

auth_a_s_vsa, auth_a_s_vsab, auth_a_b_vba,

auth_b_s_vsb, auth_b_a_vab,

auth_s_a_vas, auth_s_b_vb : protocol_id

intruder_knowledge = {a, b, s, xi, h, t, ps}

composition

session(a, b, s, xa,xb,ps, h,t)

/\ session(a, i, s, xa,xi,ps, h,t)

/\ session(i, b, s, xi,xb,ps, h,t)

/\ session(a, b, i, xa,xb,ps, h,t)

end role

% ==================== goal =====================

goal

secrecy_of sec_ua

secrecy_of sec_ub

secrecy_of sec_xa

secrecy_of sec_xb

secrecy_of sec_kab

secrecy_of sec_ks

authentication_on auth_a_s_vsa

authentication_on auth_a_s_vsab

authentication_on auth_a_b_vba

authentication_on auth_b_s_vsb

authentication_on auth_b_a_vab

authentication_on auth_s_a_vas

authentication_on auth_s_b_vb

end goal

environment()

% ================== result OFMC ==============

% OFMC

% Version of 2006/02/13

SUMMARY

SAFE

DETAILS

BOUNDED_NUMBER_OF_SESSIONS

PROTOCOL

/home/span/span/testsuite/results/3pake-AVISPA.if

GOAL

as_specified

BACKEND

OFMC

COMMENTS

STATISTICS

parseTime: 0.00s

searchTime: 0.08s

visitedNodes: 15 nodes

depth: 4 plies

% ======================= result CL-AtSe ===========================

SUMMARY

SAFE

DETAILS

BOUNDED_NUMBER_OF_SESSIONS

TYPED_MODEL

PROTOCOL

/home/span/span/testsuite/results/3pake-AVISPA.if

GOAL

As Specified

BACKEND

CL-AtSe

STATISTICS

Analysed : 3 states

Reachable : 0 states

Translation: 0.04 seconds

Computation: 0.00 seconds
